# Construction and efficacy testing of DNA vaccines containing HLA-A*02:01-restricted SARS-CoV-2 T-cell epitopes predicted by immunoinformatics: DNA vaccines containing HLA-A*02:01-restricted SARS-CoV -2 T-cell epitopes

**DOI:** 10.3724/abbs.2024039

**Published:** 2024-04-24

**Authors:** Dan Tan, Ning Kang, Yuanfei Zhu, Jia Hou, Hanqing Wang, Huijun Xu, Cheng Zu, Zixiang Gao, Mu Liu, Nannan Liu, Qiang Deng, Hongzhou Lu, Jing Liu, Youhua Xie

**Affiliations:** 1 Key Laboratory of Medical Molecular Virology (NHC & MOE & CAMS) Shanghai Institute of Infectious Diseases and Biosecurity Department of Medical Microbiology and Parasitology School of Basic Medical Sciences Shanghai Medical College Fudan University Shanghai 200031 China; 2 Shanghai Public Health Clinical Center Fudan University Shanghai 201508 China; 3 Department of Clinical Laboratory Children’s Hospital Fudan University Shanghai 201102 China; 4 National Clinical Research Centre for Infectious Diseases the Third People’s Hospital of Shenzhen The Second Affiliated Hospital of Southern University of Science and Technology Shenzhen 518112 China

**Keywords:** COVID-19, cellular immune response, CD8 ^+^ T cell, SARS-CoV-2, DNA vaccine

## Abstract

Vaccines play essential roles in the fight against the COVID-19 pandemic. The development and assessment of COVID-19 vaccines have generally focused on the induction and boosting of neutralizing antibodies targeting the SARS-CoV-2 spike (S) protein. Due to rapid and continuous variation in the S protein, such vaccines need to be regularly updated to match newly emerged dominant variants. T-cell vaccines that target MHC I- or II-restricted epitopes in both structural and non-structural viral proteins have the potential to induce broadly cross-protective and long-lasting responses. In this work, the entire proteome encoded by SARS-CoV-2 (Wuhan-hu-1) is subjected to immunoinformatics-based prediction of HLA-A*02:01-restricted epitopes. The immunogenicity of the predicted epitopes is evaluated using peripheral blood mononuclear cells from convalescent Wuhan-hu-1-infected patients. Furthermore, predicted epitopes that are conserved across major SARS-CoV-2 lineages and variants are used to construct DNA vaccines expressing multi-epitope polypeptides. Most importantly, two DNA vaccine constructs induce epitope-specific CD8 ^+^ T-cell responses in a mouse model of HLA-A*02:01 restriction and protect immunized mice from challenge with Wuhan-hu-1 virus after hACE2 transduction. These data provide candidate T-cell epitopes useful for the development of T-cell vaccines against SARS-CoV-2 and demonstrate a strategy for quick T-cell vaccine candidate development applicable to other emerging pathogens.

## Introduction

The global COVID-19 pandemic caused by severe acute respiratory syndrome coronavirus 2 (SARS-CoV-2) first emerged at the end of 2019 and eventually transitioned from the emergency phase to the endemic phase after three years [1]. Vaccines produced by both classical vaccine platforms, such as inactivated virus, recombinant viral vector and recombinant subunit vaccines, and novel platforms, most notably synthetic mRNA-based vaccines, play vital roles in ameliorating the damage caused by viruses [2]. The development and evaluation of SARS-CoV-2 vaccine candidates have generally focused on their ability to induce and boost neutralizing antibodies against the viral spike protein (S), which is responsible for receptor binding and viral entry [3]. However, immune selection by infection- and vaccination-induced antibodies resulted in significantly quicker variation in S than in response to other structural and non-structural proteins [4]. Consequently, currently licensed vaccines require regular updates to provide sufficient protection against newly emerged dominant SARS-CoV-2 variants [ 5, 6] . On the other hand, antibody titers have been shown to decrease rather rapidly in both vaccine recipients and infected patients, resulting in weakened protection against (re)infection that necessitates additional booster injections [ 7, 8] .

In addition to S-targeting neutralizing antibodies, cellular immune responses to SARS-CoV-2 are another important part of protective immunity [ 9, 10] . Unlike neutralizing antibodies, cellular responses are not limited to the spike protein and can target epitopes located in all virally encoded structural and non-structural proteins. For this reason, T-cell immunity against epitopes that are conserved across SARS-CoV-2 lineages and variants could theoretically provide significant cross-protection, even against future variants. Although some of the currently licensed S-targeting vaccines have been shown to induce T-cell responses that last longer than antibody responses [ 11, 12] , vaccine platforms with a focus on inducing T-cell responses could be expected to perform better in this regard [13]. Indeed, multiple candidate SARS-CoV -2 T-cell vaccines have been studied and are currently in various stages of development [ 14, 15] .

Specific binding between T-cell receptors (TCRs) on the surface of T cells and short peptides (usually between 8 and 14 amino acid residues) bound to class I or II major histocompatibility complexes (MHC-I/II) on the surface of antigen-presenting cells is the key step in the T-cell response [16]. These peptides represent T-cell epitopes and are produced by proteolytic antigen processing of intracellularly expressed proteins in the ER (for presentation by MHC-I) or of phagocytosed extracellular proteins in endosomes/lysosomes (for presentation by MHC-II) [17]. Epitope presentation by MHC-I and MHC-II complexes leads to the activation of CD8 ^+^ effector T cells (cytotoxic T lymphocytes, CTLs) and CD4 ^+^ helper T cells, respectively [ 16, 17] . The MHC-I complex is a heterodimer between an α chain encoded by highly polymorphic HLA-A, HLA-B and HLA-C loci in humans; H2-K, HLA-D and HLA-L loci in mice; and a β chain (β2-microglobulin/B2M) [18].

Identification and verification of immunogenic epitopes constitute the first step in T-cell vaccine development. Immunoinformatics-based T-cell epitope prediction has become an increasingly popular and important approach to epitope discovery for prophylactic, therapeutic and other related applications [ 19, 20] . Epitopes predicted *in silico* can then be verified by analyzing their recognition by T cells from infected patients. Compared to epitope screening using various forms of epitope libraries [ 21, 22] , immunoinformatics-based prediction could significantly reduce the number of epitopes to be tested and consequently reduce the time, resource and human sample costs. The predicted epitopes that pass verification will then form the basis for T-cell vaccine design, and the immunogenicity and protection efficacy of the constructed vaccine candidates will be tested in humanized animal models and human subjects. This strategy of T-cell vaccine development has also been applied to SARS-CoV-2 [23], yet published studies thus far often involved only epitopes from a limited selection of viral proteins, were limited to *in silico* analyses, or failed to provide *in vivo* efficacy testing results [24].

In the present study, we used the Immune Epitope Database (IEDB) epitope prediction platform [25] and scanned the entire SARS-CoV-2 (Wuhan-hu-1) proteome for potential CD8 ^+^ T-cell epitopes that are present in one of the most prevalent HLA-A subtypes (HLA-A*02:01) [ 26, 27] . The immunogenicity of the predicted epitopes in natural infections was tested by analyzing the recognition of chemically synthesized epitope peptides by peripheral blood mononuclear cells (PBMCs) from convalescent SARS-CoV-2 (Wuhan-hu-1)-infected patients. Furthermore, multi-epitope DNA vaccine constructs based on conserved predicted epitopes were designed and used to establish a mouse model of HLA-A*02:01-restrictedness. Epitope-specific CD8 ^+^ T-cell responses were measured to assess the immunogenicity of the vaccines, and protection efficacy was evaluated by challenging immunized mice with SARS-CoV-2 (Wuhan-hu-1) viruses following transduction with hACE2-expressing recombinant adenovirus.

## Materials and Methods

### SARS-CoV-2 sequences

For epitope prediction, the amino acid sequences of all proteins encoded by SARS-CoV-2 (Wuhan-hu-1) (GenBank accession number NC_045512) were retrieved. For the analysis of epitope conservation, corresponding sequences encoded by major lineages and variants were retrieved, including Beta (variant B.1.351/MZ433432.1), Gamma (variant P.1/MZ477859.1), Delta (variants B.1.617.2/OK091006.1, AY.3.1/ON834880.1, AY.3/OQ905725.1, AY.25.1/ON834811.1, AY.25/ON834805.1, AY.39.1/ON834816.1, AY.39/ON834809.1, AY.44/ON834818.1, AY.47/ON834815.1, AY.100/ON834822.1, AY.103/ON834807.1), and Omicron (variants B.1.1.529/OM570283.1, BA.2.3/OR352440.1, BA.2.9/OR325311.1, BA.2.12/OP790345.1, BA.2.18/OR325322.1, BA.2.37/OR325193.1, BA.2/ON834972.1, BA.4/OR325403.1, BA.5/OR277772.1).

### Epitope prediction and peptide synthesis

Sequences of all SARS-CoV-2 (Wuhan-hu-1)-encoded proteins were submitted to the Immune Epitope Database (IEDB) ( http://tools.iedb.org/mhci/) for the prediction of HLA-A*02:01-restricted epitopes with a length of 14 amino acids using the NetMHCpan 4.1 EL algorithm [28]. Epitopes with “percentile rank” values equal to or less than 2 were selected for further analysis. A total of 604 epitopes meeting these criteria were obtained and sent for chemical synthesis (GenScript, Shanghai, China). Only 524 peptides were successfully synthesized and obtained at a purity of 85% or greater ( Supplementary Tables S1 and S2). Peptides were dissolved in DMSO (Sigma, St Louis, USA) as 1 mg/mL stock solutions and stored at ‒80°C until use.

### PBMC samples

PBMCs were prepared via Percoll (Sigma) fractionation from peripheral blood samples collected from 20 HLA-A*02:01-homozymous SARS-CoV-2 (Wuhan-hu-1)-infected convalescent patients admitted to the Shanghai Public Health Clinical Center between January and February of 2020. Two HLA-A*02:01-homozymous PBMC samples prepared from healthy blood donors collected prior to the end of 2019 were purchased from AllCells Biotech (Shanghai, China). HLA-A PCR-sequencing-based typing (PCR-SBT) was performed by BGI (Shenzhen, China). All PBMC samples were cultured in R10 media (RPMI 1640 medium supplemented with 10% fetal bovine serum, 100 U/mL penicillin, 100 μg/mL streptavidin, 2 mM L-glutamine, and 20 mM HEPES; Thermo Fisher Scientific, Waltham, USA).

### Epitope peptide stimulation and IFN-γ ELISpot assay

The peptides were grouped into 26 pools containing 20 or 24 peptides ( Supplementary Table S1) for the initial assay of recognition by patient PBMCs. PMBCs (2×10 ^5^ cells in R10 media) were stimulated with an epitope peptide at a final concentration of 5 μg/mL or with a peptide pool containing each peptide at 5 μg/mL. Stimulation was performed in triplicate for 24 h, and IFN-γ production was measured using an ELISpot assay [ELISpot Pro: Human IFN-γ (ALP); Mabtech, Stockholm, Sweden] according to the manufacturer’s instructions. Mock-stimulated and anti-CD3-stimulated cells were used as negative and positive controls, respectively, and spots were counted using an iSpot reader (AID, Strassberg, Germany). The results are presented as the relative number of spot-forming cells (relative SFC) relative to that of the negative control: [SFC (peptide or peptide pool)‒SFC (mock)]/SFC (mock). A relative SFC≥3 was considered to indicate that the PBMC sample displayed positive recognition of and response to the corresponding peptide (pool).

### DNA vaccine design, construction, and preparation

The selected predicted epitopes, as indicated, were concatenated using an EAAAK linker and fused at the N-terminus with a FLAG tag and at the C-terminus with a promiscuous CD4 ^+^ T-cell epitope (PADRE) [29] through a GPGPG linker. The coding sequences of the designed multiepitope polypeptides were chemically synthesized (Tsingke, Beijing, China), cloned, and inserted into a CMV promoter-driven mammalian expression vector (Beyotime, Shanghai, China). The sequences of the obtained constructs were confirmed by automatic sequencing (Tsingke). Endotoxin-free plasmid DNA for immunization was prepared using an EndoFree Plasmid kit from Qiagen (Hilden, Germany). For verification of multi-epitope polypeptide expression, HEK293T cells were transfected with the DNA vaccine constructs and subjected to immunofluorescence and western blot analyses using an anti-FLAG monoclonal antibody (Sigma) as the primary antibody and Alexa 488- and HRP-labelled goat-anti-mouse IgG (Thermo Fisher Scientific) as the secondary antibodies, respectively.

### Mouse DNA vaccination

Male 6- to 8-week-old B-HLA-A2.1 mice were purchased from Biocytogen (Beijing, China) and housed in the Laboratory Animal Center of Shanghai Medical College at Fudan University. An intramuscular injection of 25 μg of plasmid DNA was performed on the left thigh of the mice, followed by electroporation using CELLECTRA-3P (Inovio, San Diego, USA) with the parameter OpBlock 0079. Two doses of DNA vaccines separated by 3 weeks were given, and immunized mice were randomly selected for immune response analysis or virus challenge as described below.

### Assay of the epitope-specific CD8 ^+^ T-cell response in DNA-vaccinated mice

Mice were sacrificed two weeks after the second dose of the DNA vaccine. Splenocytes were prepared from excised spleens and stimulated with the indicated epitope peptide at a final concentration of 5 μg/mL for 24 h. IFN-γ production was analyzed using intracellular IFN-γ staining following a published protocol [30]. The cells were also stained with Fixable Viability Stain 780 and for surface markers with FITC-conjugated anti-CD3 and PerCP-Cy5.5-conjugated anti-CD8 antibodies (BD Pharmingen, Franklin Lakes, USA). Mock-stimulated and PMA (100 ng/mL) plus ionomycin (1 μg/mL) (Univ, Shanghai, China)-stimulated splenocytes were processed in parallel as negative and positive controls, respectively. Stained samples were analyzed using a BD LSR Fortesa flow cytometer, and acquired events were processed using FlowJo. The results are presented as a percentage of IFN-γ ^+^ cells within the live CD3 ^+^CD8 ^+^ T-cell population in each sample.

### SARS-CoV-2 challenge of vaccinated mice

SARS-CoV-2 mouse challenge experiments were performed in the BSL-3 laboratory of Fudan University. Susceptibility to SARS-CoV-2 was induced in immunized B-HLA-A2.1 mice by transduction with recombinant adenovirus serotype 5 expressing human ACE2 (rAd5-hACE2; HanBio, Shanghai, China). Eight days after the second dose of DNA, the mice were anaesthetized with 2.5×10 ^9^ geq (genome equivalent) of rAd5-hACE2 in 50 μL of PBS through nasal dripping. Five days later, the mice were similarly administered with SARS-CoV-2 (Wuhan-hu-1) virus (1.5×10 ^5^ TCID _50_) in 50 μL of PBS and monitored for 5 days before sacrifice for tissue collection.

The left lungs were fixed in 4% paraldehyde, and paraffin-embedded tissue sections were prepared with Pathology slicer RM2016 (Leica, Shanghai, China) and subjected to H&E staining (Aifang Biological, Changsha, China), followed by image analysis using the K-Viewer (1.7.0.29) software (KFBIO, Hangzhou, China). The right lungs were preserved in Trizol Reagent (Thermo Fisher), and total RNA was extracted from 20 mg of lung tissue. SARS-CoV-2 RNA was quantified via reverse transcription followed by quantitative real-time PCR (RT-qPCR) using a primer set targeting the viral N protein: forward primer: 5′-GGGGAACTTCTCCTGCTAGAAT-3′; and reverse primer: 5′-CAGACATTTTGCTCTCAAGCTG-3′.

### Ethics statement

The use of peripheral blood samples from human subjects in this work was reviewed and approved by the Ethics Committee of the Shanghai Public Health Clinical Center (No. YJ-2020-S021-01). Written informed consent was obtained from every participant. The use of mice and relevant procedures were approved by the Animal Ethics Committee of School of Basic Medical Sciences, Fudan University (No. FE21113).

## Results

### Immunoinformatics-based prediction of HLA-A*02:01-restricted T-cell epitopes in the SARS-CoV-2 proteome

To screen and test potential CD8 ^+^ T-cell epitopes of SARS-CoV-2, all ORFs encoded by the original Wuhan-Hu-1 isolate were submitted to the IEDB for the prediction of 14 aa epitopes presentable by HLA-A*02:01, one of the most prevalent HLA-A subtypes. A total of 604 epitopes were predicted (percentile rank<2; Supplementary Table S1), which are located in nearly all structural and non-structural proteins and encompassed ~30%–90% of the length of the corresponding protein ( Supplementary Table S2). All 604 predicted epitopes were subjected to chemical synthesis, but only 524 were successfully synthesized and purified to >85% purity ( Supplementary Tables S1 and S2). Only these 524 epitopes were used in subsequent analyses.

### Predicted HLA-A*02:01-restricted epitope peptides stimulated IFN-γ production by PBMCs from convalescent patients

**Figure 1:**
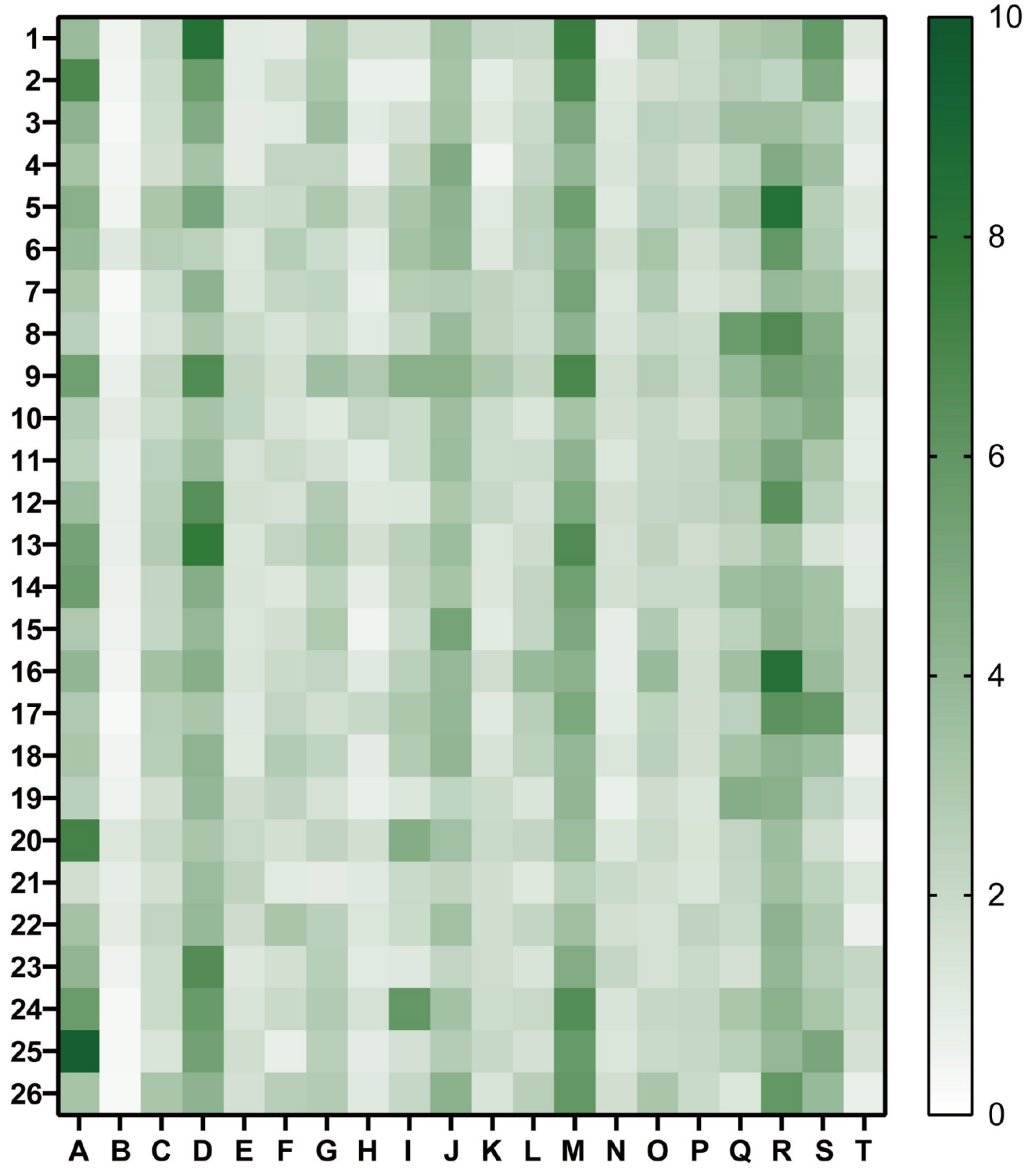
Figure 1 IFN-γ production by convalescent patient PBMCs stimulated with pools of epitope peptides PBMCs (2×10 5 cells) derived from 20 HLA-A*02:01-homozygous SARS-CoV-2 (Wuhan-hu-1)-infected convalescent patients (A-T) were stimulated in triplicate with the indicated epitope peptide pools (1‒26; Supplementary Table S1) at 5 μg/mL of each peptide for 24 h. IFN-γ-producing cells were measured using ELISpot, and mock-stimulated and anti-CD3-stimulated cells were used as negative and positive controls, respectively. The relative number of spot-forming cells is shown using the indicated color scale. A relative SFC≥3 was considered a positive stimulation result.

To test whether the predicted epitopes are immunogenic in natural infections, PBMCs were isolated from whole blood samples of 20 HLA-A*02:01-homozygous convalescent patients (labelled A-T) infected with the Wuhan-Hu-1 isolate at the start of 2020 ( Supplementary Table S3) and subjected to peptide-stimulated IFN-γ production analysis. Due to the limited number of PBMCs available, 524 epitope peptides were first pooled into 26 pools containing 20 or 24 peptides ( Supplementary Table S1). Convalescent patient-derived PBMCs were stimulated with each pool containing 5 μg/mL of each peptide, and the IFN-γ ELISPOT assay was used to measure IFN-γ production. As shown in Figure 1, the majority of the PBMC samples displayed IFN-γ production after stimulation with multiple peptide pools, most notably PBMCs from patients A, D, M, and R. To identify the individual peptide(s) responsible for the induction of IFN-γ production, the PBMC samples were then stimulated with the 1 or 3 (depending on the amount of PBMCs available) highest IFN-γ-stimulating pool(s) in Figure 1, and the IFN-γ ELISPOT assay identified 33 epitope peptides that are recognized by patient PBMCs, which are located in the M, N and multiple nonstructural proteins ( Table 1).

**Table:** **Table 1** Epitopes recognized by PBMCs from convalescent patients

Epitope	ORF	Start	End	Protein	Sequence	Rank*	Patient
ORF1ab2787-4200aa-25	1ab	3470	3483	nsp5	WLYAAVINGDRWFL	0.27	K
ORF1ab1-1400aa-22	1ab	600	613	nsp2	YITGGVVQLTSQWL	0.29	M
ORF1ab2787-4200aa-27	1ab	3482	3495	nsp5	FLNRFTTTLNDFNL	0.29	K
ORF1ab2787-4200aa-28	1ab	3084	3097	nsp4	LFLMSFTVLCLTPV	0.29	K
ORF1ab2787-4200aa-29	1ab	3047	3060	nsp4	IVAGGIVAIVVTCL	0.29	K
ORF1ab2787-4200aa-31	1ab	4039	4052	nsp8	KLDNDALNNIINNA	0.31	K
ORF1ab1-1400aa-26	1ab	641	654	nsp2	FLRDGWEIVKFIST	0.35	M
ORF1ab2787-4200aa-38	1ab	3886	3899	nsp7	KLWAQCVQLHNDIL	0.35	K
ORF1ab2787-4200aa-42	1ab	3013	3026	nsp4	SLPGVFCGVDAVNL	0.37	K
ORF1ab2787-4200aa-44	1ab	3678	3691	nsp6	KLKDCVMYASAVVL	0.39	K
ORF1ab1-1400aa-38	1ab	26	39	nsp1	VLVRGFGDSVEEVL	0.46	M
ORF1ab2787-4200aa-56	1ab	3898	3911	nsp7	ILLAKDTTEAFEKM	0.5	K
M-9	M	15	28	M	KLLEQWNLVIGFLF	0.51	O
ORF1ab2787-4200aa-62	1ab	2872	2885	nsp4	TILRTTNGDFLHFL	0.53	H
ORF1ab4397-5796aa-36	1ab	5751	5764	nsp13	RLMKTIGPDMFLGT	0.58	K
ORF1ab1-1400aa-47	1ab	209	222	nsp2	GKASCTLSEQLDFI	0.62	M
ORF1ab2787-4200aa-89	1ab	3546	3559	nsp5	GSALLEDEFTPFDV	0.71	K
ORF1ab2787-4200aa-90	1ab	3748	3761	nsp6	VTTVMFLARGIVFM	0.71	K
ORF1ab2787-4200aa-94	1ab	3364	3377	nsp5	YKFVRIQPGQTFSV	0.73	K
ORF1ab2787-4200aa-4	1ab	3634	3647	nsp6	AFLCLFLLPSLATV	0.76	O
ORF1ab4187-4405aa-3	1ab	4233	4246	nsp9	GLNNLNRGMVLGSL	0.76	K
ORF1ab1-1400aa-60	1ab	1382	1395	nsp3	AEETRKLMPVCVET	0.78	M
ORF1ab1-1400aa-72	1ab	1299	1312	nsp3	VVIPTKKAGGTTEM	0.84	M
ORF1ab1-1400aa-82	1ab	1117	1130	nsp3	VVGPNVNKGEDIQL	0.93	D
ORF1ab1-1400aa-84	1ab	1045	1058	nsp3	IVEEAKKVKPTVVV	0.93	M
ORF1ab2787-4200aa-120	1ab	3912	3925	nsp7	VSLLSVLLSMQGAV	0.93	O
ORF1ab4187-4405aa-6	1ab	4224	4237	nsp9	KVKYLYFIKGLNNL	0.95	K
ORF1ab2787-4200aa-125	1ab	3268	3281	nsp5	KMAFPSGKVEGCMV	0.97	K
ORF1ab2787-4200aa-130	1ab	3462	3475	nsp5	TITVNVLAWLYAAV	0.99	K
N28274-29533-2	N	217	230	N	AALALLLLDRLNQL	1.4	O
ORF1ab1-1400aa-4	1ab	1285	1298	nsp3	YIVGDVVQEGVLTA	1.5	M
ORF1ab2787-4200aa-10	1ab	3705	3718	nsp6	ARRVWTLMNVLTLV	1.5	K
ORF1ab2787-4200aa-11	1ab	2956	2969	nsp4	LMDGSIIQFPNTYL	1.5	O

*The percentile rank is calculated with the IEDB prediction algorithm.

To rule out nonspecific and cross-reactive stimulation, all 524 epitope peptides were also used individually to stimulate two commercially purchased HLA-A*02:01-homozygous PBMC samples collected prior to the pandemic from healthy donors. Only one epitope peptide, which was not among the 33 identified above, produced a positive result against one PBMC sample in the IFN-γ ELISPOT assay ( Supplementary Table S4). Although this epitope is highly conserved among Sarbecoviruses (data not shown), the lack of response to other tested SARS-CoV-2 epitopes suggests that this response was unlikely to be a result of prior exposure to related virus(es). Collectively, these data indicated that HLA-A*02:01-restricted epitopes predicted by immunoinformatics could indeed be immunogenic in natural SARS-CoV-2 infections of HLA-A*02:01 patients.

### Design of DNA vaccines based on predicted SARS-CoV-2 T-cell epitopes

**Figure 2:**
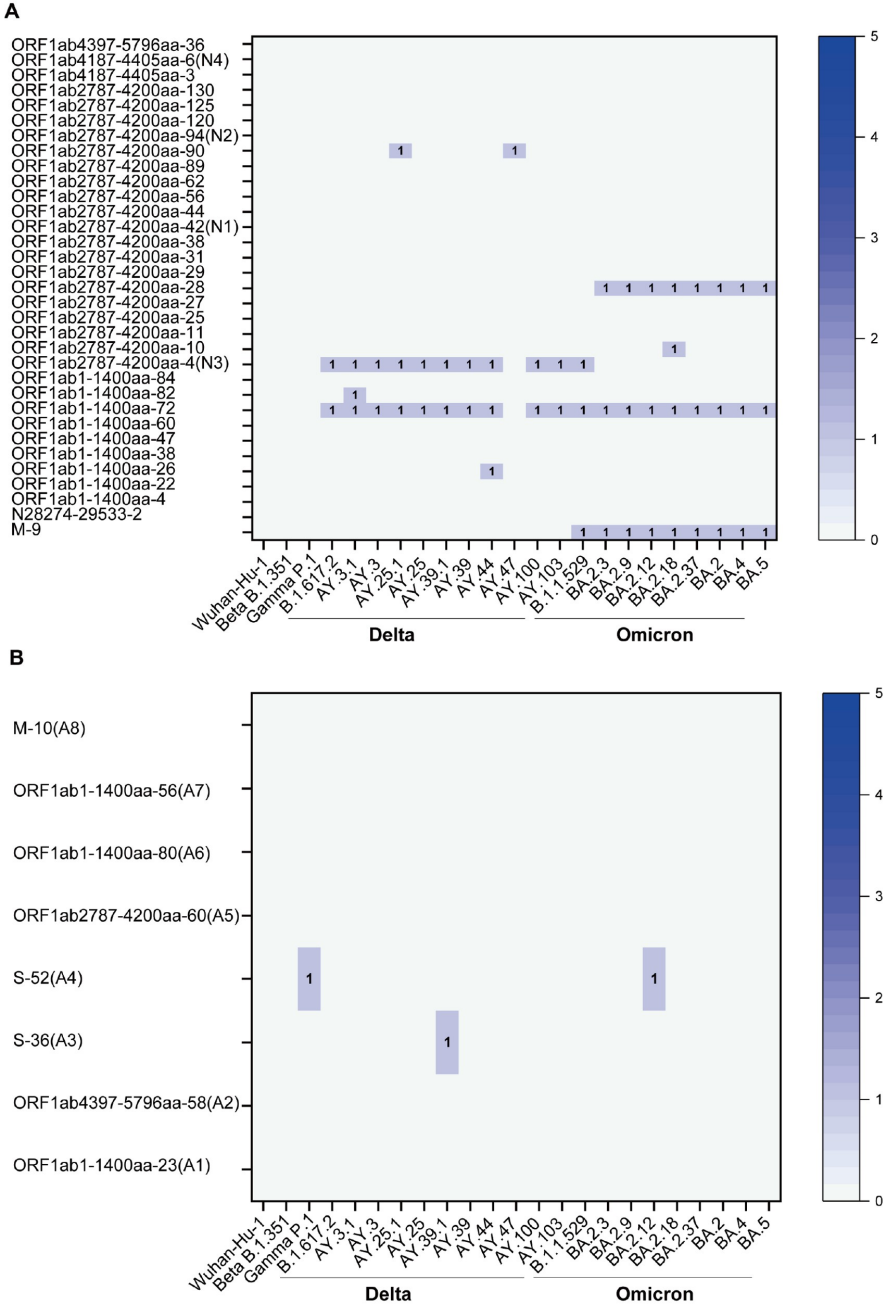
Figure 2 Conservation of epitope sequences in major SARS-CoV-2 lineages and strains (A) Epitopes that individually stimulated IFN-γ production in convalescent patient PBMCs (see Table 1) were analyzed for sequence conservation among other major SARS-CoV-2 lineages and strains as indicated. The number of different residues within the 14 aa epitope compared to the original Wuhan-Hu-1 isolate is shown (0 is shown as a blank for clarity). Epitopes selected from DNA vaccine design (N1–N4) are indicated. (B) The additional epitopes A1–A8 not in Table 1 were also selected for DNA vaccine design.

Based on the predicted and partially verified HLA-A*02:01-restricted SARS-CoV -2 T-cell epitopes, we designed DNA vaccine constructs expressing multiple epitopes. Because the virus has undergone significant variation and evolution since the start of the pandemic, the sequence conservation of predicted epitopes was first analyzed against all major SARS-CoV-2 lineages and variants. As shown in Figure 2A, all 33 epitopes identified above are highly conserved, and 4 of these (designated N1-N4) were selected for vaccine design. Additionally, since patients with similar HLA-A genetic backgrounds responded differently to infection by the same virus with regard to T-cell responses ( Figure 1 and Table 1), 8 highly conserved epitopes (designated A1‒A8, Figure 2B) that were not recognized by either patient or healthy donor PBMCs were also selected for testing.

**Figure 3:**
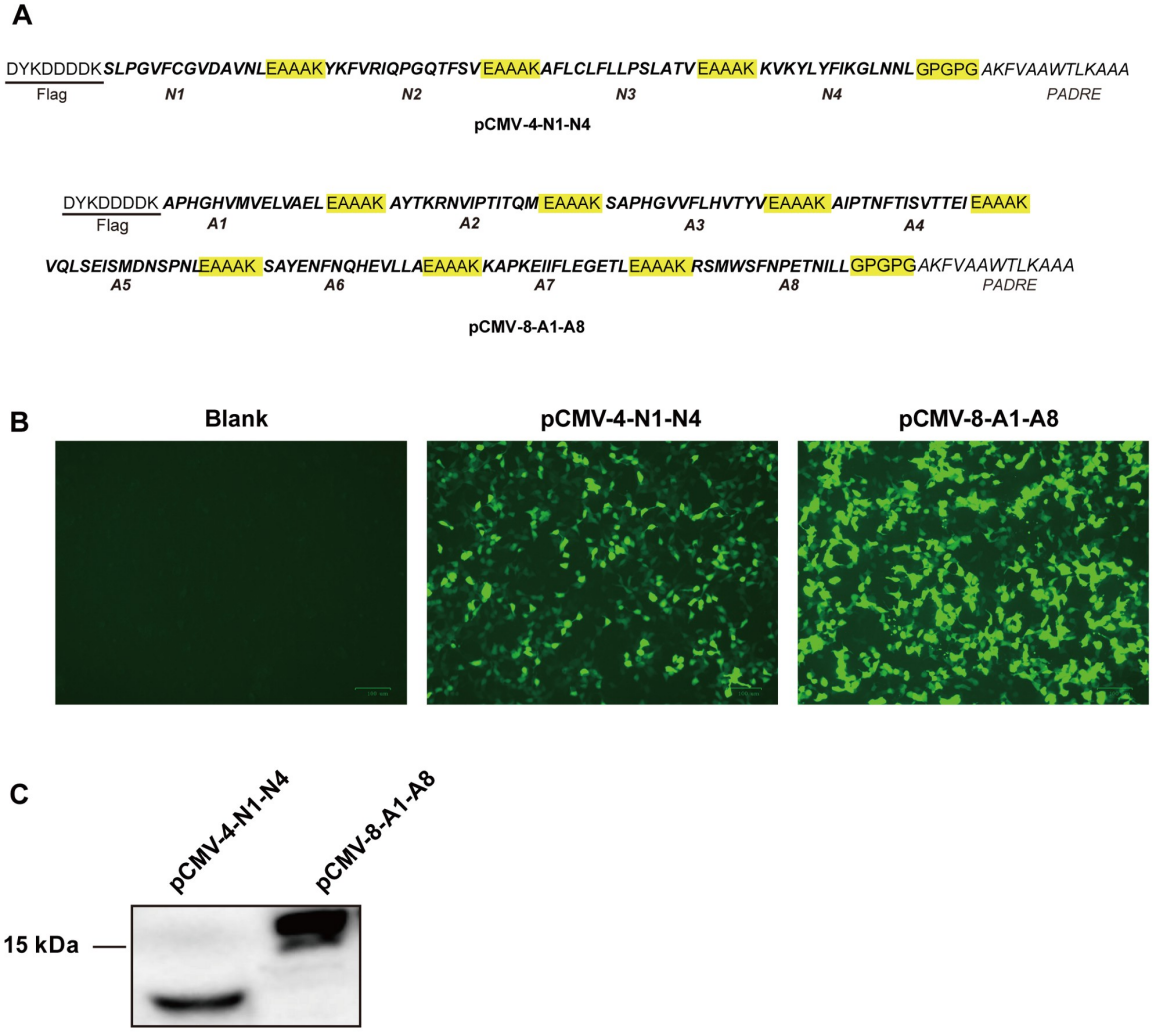
Figure 3 Multi-epitope DNA vaccine design and expression analysis (A) Amino acid sequences of multi-epitope polypeptides encoded by the pCMV-4-N1-N4 (top) and pCMV-8-A1-A8 (bottom) DNA vaccine constructs. The plasmids were transfected into HEK293T cells, and FLAG-tagged multi-epitope polypeptide expression was analyzed using an anti-FLAG monoclonal antibody via immunofluorescence (B) and western blot (C) analysis.

For DNA vaccine design, the amino acid sequences of N1-N4 and A1-A8 were joined together through an EAAAK linker and fused at the N-terminus with a FLAG tag ( Figure 3A). A nonnatural promiscuous CD4 ^+^ T-cell epitope (PADRE) was also fused to the C-terminus through a GPGPG linker to provide potential adjuvant effects. The corresponding cDNA sequences were chemically synthesized and inserted into a CMV promoter-driven mammalian expression vector to create pCMV-4-N1-N4 and pCMV-8-A1-A8, respectively. The expression of FLAG-tagged multi-epitope polypeptide was tested by transfecting HEK293T cells with plasmids and subjecting the transfected cells to immunofluorescence and western blot analyses. As shown in Figure 3B,C, FLAG-tagged proteins were detectable in transfected cells for both pCMV-4-N1-N4 and pCMV-8-A1-A8, and the apparent molecular weights of the bands detected by western blot analysis matched the calculated molecular weights of the corresponding constructs. It was therefore clear that these DNA vaccine plasmids were capable of expressing the multi-epitope polypeptide as designed.

### DNA vaccines based on predicted epitopes induced CD8 ^+^ T-cell responses in HLA-A*02:01 transgenic mice

To test DNA vaccines based on predicted HLA-A*02:01-restricted SARS-CoV -2 T-cell epitopes, a mouse model of human HLA-A*02:01 restrictedness (B-HLA-A2.1) was used. In B-HLA-A2.1 mice, the endogenous mouse B2m gene has been replaced by the human B2m gene, and human HLA-A*02:01 epitope-binding domains have been fused to other domains of the mouse H2D gene. Only the expression and function of transgenic HLA-A*02:01, but not endogenous murine H2D ^b^, could be detected in these mice.

**Figure 4:**
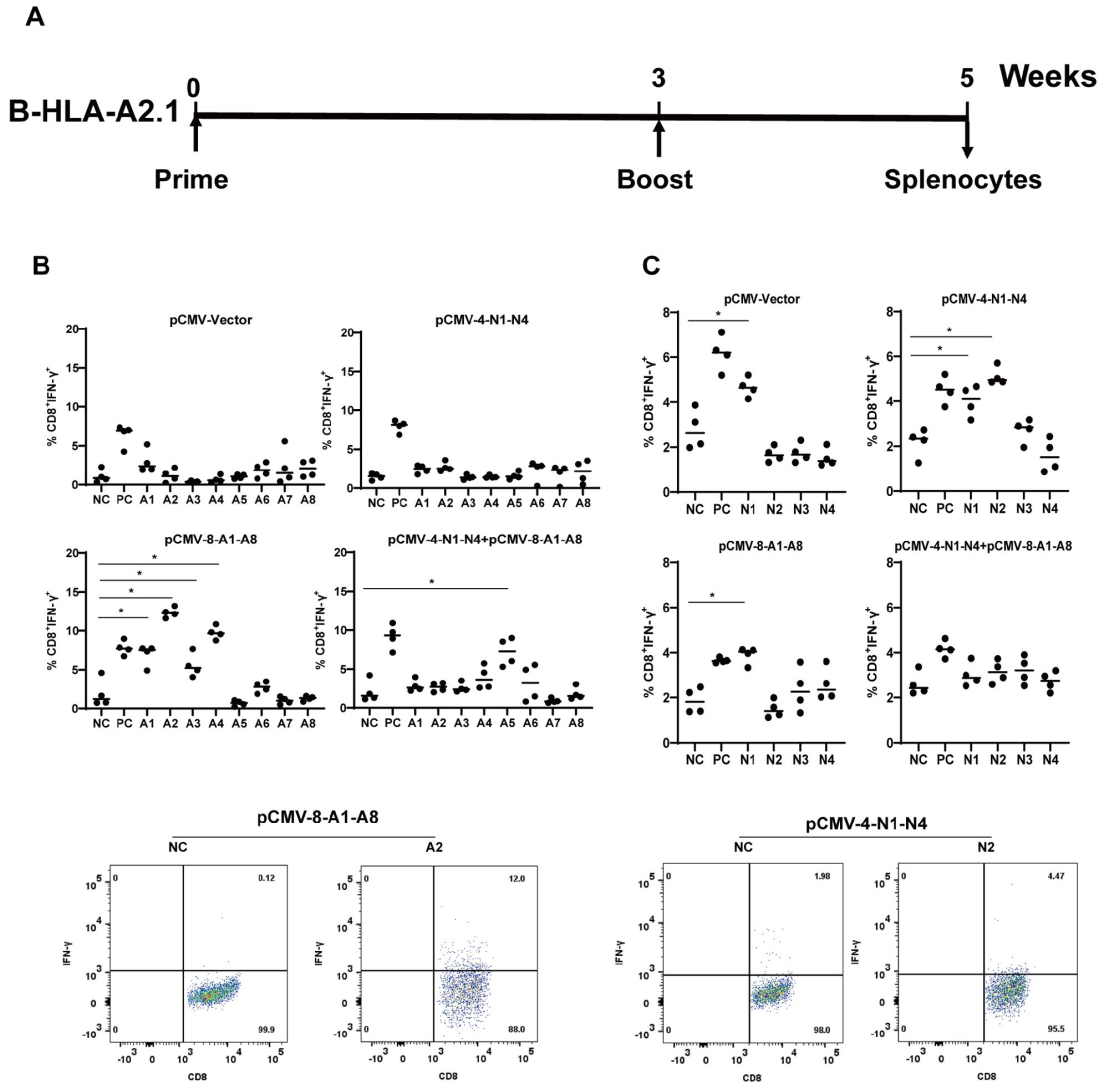
Figure 4 Epitope-specific CD8 ^+^ T-cell response in DNA vaccine-immunized B-HLA-A2.1 mice (A) Vaccination schedule. B-HLA-A2.1 mice were subjected to two doses of intramuscular injection of 25 μg plasmid DNA separated for 3 weeks and sacrificed 2 weeks after boost injection for splenocyte preparation. Splenocytes (1×10 6 cells) from mice receiving the indicated plasmids were stimulated with A1-A8 (B) or N1-N4 (C) epitope peptides (5 μg/mL) and subjected to viability staining, surface CD3 and CD8 staining, and intracellular IFN-γ staining. Mock-stimulated and PMA (100 ng/mL) plus ionomycin (1 μg/mL)-stimulated splenocytes were processed in parallel as negative (NC) and positive (PC) controls, respectively. The stained cells were analyzed by flow cytometry, and the percentages of IFN-γ + cells within the live CD3 +CD8 + T-cell population are plotted (top). Peptide-stimulated IFN-γ + cell percentages were compared against NC values obtained using the same splenocytes, and statistical significance was calculated using two-way ANOVA. Representative flow cytometry dot plots indicating positive peptide-stimulated IFN-γ production are shown (bottom). * P<0.05.

B-HLA-A2.1 mice were intramuscularly injected with 25 μg of empty vector, pCMV-4-N1-N4, pCMV-8-A1-A8, or both constructs (12.5 μg each) and boosted 3 weeks later with the same injections ( Figure 4A). Two weeks after boost injection, the mice were sacrificed, and splenocytes were prepared and stimulated with the N1-N4 and A1-A8 epitope peptides. The percentage of IFN-γ-producing cells among live CD3 ^+^CD8 ^+^ T cells among the peptide-stimulated splenocytes was then measured via intracellular IFN-γ staining and flow cytometry. As shown in Figure 4B,C, pCMV-4-N1-N4 immunization induced a detectable N2-specific CD8 ^+^ T-cell response, while pCMV-8-A1-A8 immunization induced detectable responses specific for the A1, A2, A3 and A4 epitopes. In mice immunized with both pCMV-4-N1-N4 and pCMV-8-A1-A8, however, a specific response was only detectable for the A5 epitope. No specific responses were detected in vector-immunized mice except towards the N1 epitope, which also non-specifically stimulated IFN-γ production by CD8 ^+^ T cells from pCMV-8-A1-A8-immunized mice ( Figure 4B,C). These data demonstrated that immunization with the designed DNA vaccine constructs was capable of inducing CD8 ^+^ T-cell responses against at least some of the included epitopes. In other words, these predicted HLA-A*02:01-restricted epitopes were processed and presented by the HLA-A*02:01 complex in this model.

### DNA vaccine immunization protected mice from SARS-CoV-2 challenge

**Figure 5:**
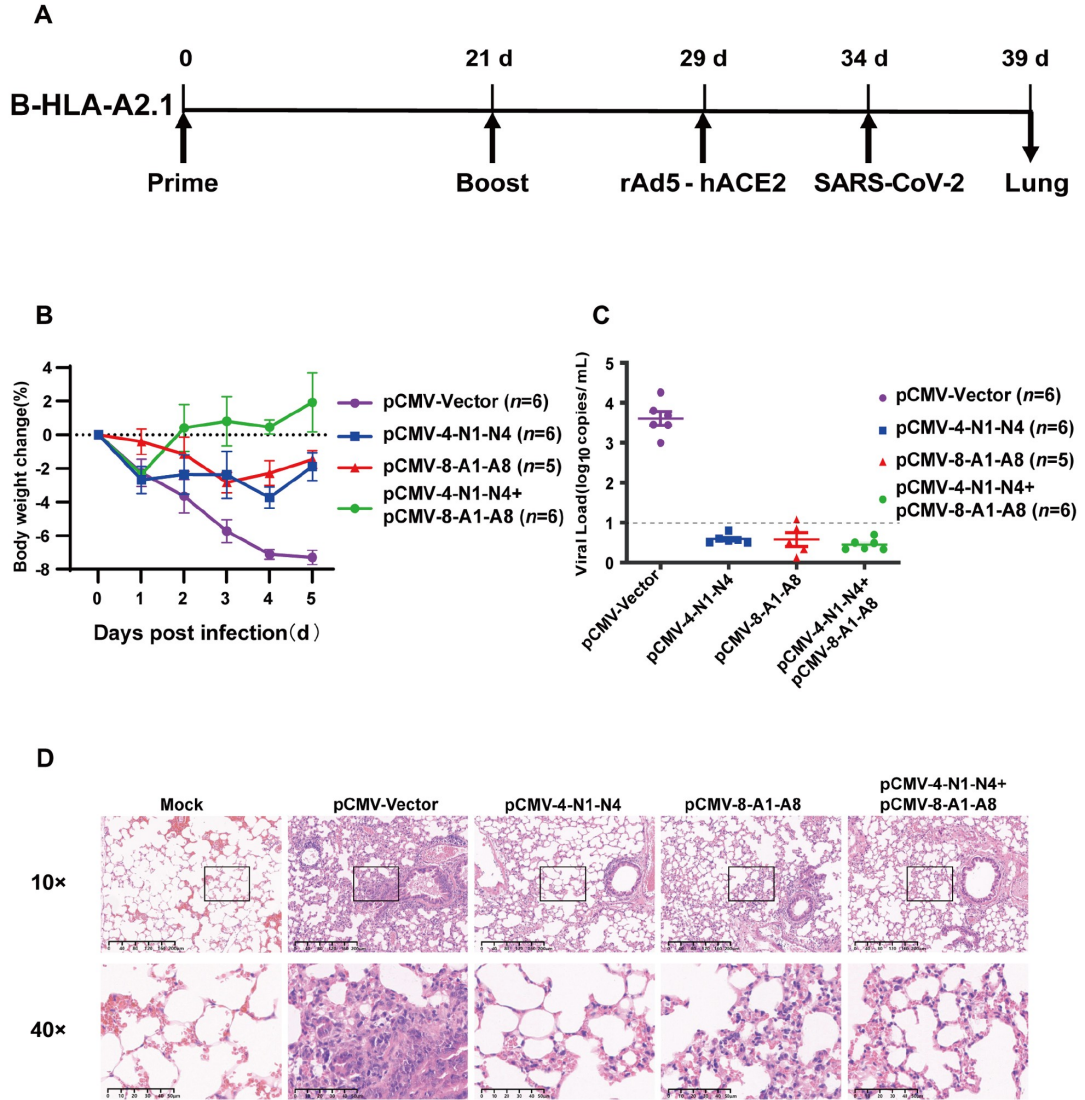
Figure 5 DNA vaccine protects B-HLA-A2.1 mice from SARS-CoV-2 challenge (A) Vaccination and challenge schedule. B-HLA-A2.1 mice received two doses of intramuscular injection of 25 μg plasmid DNA separated by 3 weeks, and 8 days after the boost injection, they were transduced with 2.5×10 9 geq (genome equivalent) of rAd5-hACE2 in 50 μL of PBS through nasal dripping, and 5 days later, they were challenged with SARS-CoV-2 (1.5×10 5 TCID 50) in 50 μL of PBS through nasal dripping. Mice were monitored for 5 days before being sacrificed for lung tissue excision. (B) Postchallenge body weight changes. (C) Measurement of SARS-CoV-2 RNA in lung tissue by RT-qPCR. The dotted line represents the lower limit of detection. (D) H&E staining of lung tissue sections from challenged mice. Representative images from each group are shown.

We tested whether DNA vaccine immunization offers immune protection against SARS-CoV-2 infection. For this purpose, B-HLA-A2.1 mice vaccinated as described above were intranasally inoculated with recombinant adenovirus expressing human ACE2 (rAd5-hACE2) 8 days after boost injection to increase SARS-CoV-2 susceptibility in the respiratory tract ( Figure 5A). Five days after rAd5-hACE2 infection, the mice were nasally challenged with Wuhan-Hu-1 virus, monitored for 5 days, and then sacrificed for lung tissue collection. Compared with vector-injected mice, mice that received single or mixed DNA vaccines displayed less marked postchallenge decreases in body weight ( Figure 5B). Quantification of the SARS-CoV-2 viral load in lung samples also revealed that lungs from vector-immunized control mice contained easily detectable amounts of viral RNA, whereas lungs from mice immunized with pCMV-4-N1-N4, pCMV-8-A1-A8, or both contained no detectable SARS-CoV-2 RNA ( Figure 5C). Similarly, histochemistry analysis of lung tissue sections revealed marked disruption of alveolar structures in vector-immunized mice but not in mice receiving multiepitope DNA vaccines ( Figure 5D). Taken together, these observations demonstrated that immunization with DNA plasmids expressing polypeptides consisting of predicted HLA-A*02:01-restricted SARS-CoV-2 epitopes induced protection against virus challenge in this mouse model of the HLA-A*02:01-restricted immune context.

## Discussion

Immunoinformatics-based T-cell epitope prediction algorithms such as those implemented by the IEDB allow *in silico* prediction of potentially immunogenic epitopes of specified length(s) and MHC specificities. In this study, we selected one of the most prevalent HLA-A subtypes, namely, HLA-A*02:01 [ 26, 27] , and selected an epitope length of 14 amino acid residues for prediction ( Supplementary Table S1). It should be noted that it is possible for an epitope peptide or different parts of the same longer epitope peptide to be presentable by different MHC type/subtype molecules. Some of the epitopes studied in this work also had high immunogenicity potential when other HLA-A subtypes were used for epitope prediction (data not shown). Testing such epitopes using relevant human PBMCs and corresponding mouse models similar to B-HLA-A2.1 might result in the identification of SARS-CoV -2 T-cell epitopes with broader HLA-A specificities.

Immune reactions to natural infection can vary significantly from person to person, even when they share similar MHC polymorphism patterns. This was observed when PBMCs from HLA-A*02:01-homozygous convalescent COVID-19 patients were stimulated with the same panel of pooled epitope peptides and analyzed for IFN-γ production ( Figure 1). Due to the limited number of PBMCs available, however, we were not able to perform further experiments to prove that the observed positive reactions were mediated through and only through HLA-A*02:01-specific presentation. Nevertheless, the remaining PBMC samples enabled us to identify 33 individual epitope peptides within these pools that stimulated IFN-γ production by patient PBMCs ( Table 1). It is highly possible that by performing the same experiments on PBMCs from more convalescent patients, the additional epitopes predicted here might also be immunogenic in natural infections and consequently warrant further verification.

DNA vaccine constructs expressing polypeptides harboring multiple epitopes allowed quick testing of epitope immunogenicity in animal models. Data obtained in B-HLA-A2.1 mice using the two constructs containing epitopes conserved across major SARS-CoV-2 lineages and variants ( Figures 2 and 3) demonstrated that at least some of the epitopes were immunogenic in this model of HLA-A*02:01-restrictedness ( Figure 4). However, additional and more detailed experiments, such as HLA-A blocking or tetramer-based analysis, are needed to confirm that the observed peptide-specific CD8 ^+^ T-cell responses may actually be mediated by HLA-A*02:01 presentation of the relevant epitopes. On the other hand, apparently, only some of the epitopes contained in the DNA vaccine constructs induced corresponding CD8 ^+^ T-cell responses in immunized mice. This might be caused either by low immunogenicity of the epitope(s) *in vivo* or by inefficient and/or incorrect processing of the expressed polypeptide in antigen-presenting cells. Low immunogenicity might be improved by increasing the dosage or including more optimized adjuvants in the vaccines, whereas modification and optimization of the construct design with regard to factors such as linker selection and length variation would be required to address the processing problem.

Recombinant adenovirus-mediated transduction of hACE2 into the respiratory tract of B-HLA-A2.1 mice allowed us to test the protective effects of the DNA vaccine constructs without having to perform tedious crossing between B-HLA-A2.1 and hACE2-transgenic mice and selection of homozygotes. Protection against SARS-CoV-2 challenge in vaccinated mice manifested as mild weight loss post infection and nearly no detectable virus in lung tissue at 5 d.p.i., coupled with much less marked disruption of alveolar structures ( Figure 5). Due to biosafety limitations, it was impossible to analyze the cellular immune responses in these challenged mice, yet since these mice were immunized in parallel with the mice shown in Figure 4, it could be safely argued that similar epitope-specific CD8 ^+^ T-cell responses were also induced in these challenged mice and likely contributed to the observed protection from SARS-CoV-2 challenge. This clearly needs to be confirmed with further experiments. In addition, although the epitopes included in the tested DNA vaccine constructs are highly conserved across major lineages and variants ( Figure 3), challenge with these variant viruses would be required to confirm whether vaccination-induced cellular responses indeed offer cross-protection.

In summary, immunoinformatics-based T-cell epitope prediction followed by epitope screening using PBMCs from convalescent patients allowed the identification of HLA-A*02:01-restricted SARS-CoV -2 T-cell epitopes that are immunogenic in natural infections. Furthermore, immunogenicity and efficacy testing of DNA vaccines containing identified epitopes revealed that these epitopes could also be immunogenic during vaccination and, more importantly, induce epitope-specific CD8 ^+^ T-cell responses and protection against SARS-CoV-2 challenge. The epitopes identified and tested in this work represent additional previously described T-cell epitopes of SARS-CoV-2, and together, they will support the future design and development of vaccines aimed at stimulating better T-cell responses in humans. Moreover, the approach to T-cell vaccine development demonstrated here could also be applied in the initial response to other novel pathogens that might emerge in the future as a complement to other classical and novel approaches to fast vaccine development.

## Supporting information

23633Supplementary_Tables

## Supplementary Data

Supplementary data is available at *Acta Biochimica et Biophysica Sinica* online.
