# Supplementary material for: Construction and efficacy testing of DNA vaccines containing HLA-A*02:01-restricted SARS-CoV-2 T-cell epitopes predicted by immunoinformatics: DNA vaccines containing HLA-A*02:01-restricted SARS-CoV -2 T-cell epitopes
Source: Acta Biochim Biophys Sin (Shanghai). 2024 Apr 24;56(7):986–96. doi: 10.3724/abbs.2024039 (PMC11322877; doi:10.3724/abbs.2024039)
Supplement: 23633Supplementary_Tables [file 23633Supplementary_Tables.pdf]

Supplementary Table S1. IEDB-predicted HLA-A*02:01-restricted $CD8^+$ epitopes

Epitope	Start in ORF	End in ORF	Protein	Sequence	Rank	Pool
ORF1ab1-1400aa-7	1257	1270	nsp3	IDINGNLHPDSATL	0.21	/
ORF1ab2787-4200aa-19	3197	3210	nsp4	VLLPLTQYNRYLAL	0.21	8
ORF1ab2787-4200aa-20	3091	3104	nsp4	VLCLTPVYSFLPGV	0.21	14
ORF1ab4397-5796aa-9	5563	5576	nsp13	TLVPQEHYVRITGL	0.21	21
ORF1ab1-1400aa-8	1138	1151	nsp3	FNQHEVLLAPLLSA	0.22	/
ORF1ab1-1400aa-9	1278	1291	nsp3	FLKKDAPYIVGDVV	0.22	2
ORF1ab1387-2800aa-12	2780	2793	nsp4	FLFVAAIFYLITPV	0.22	/
ORF1ab4397-5796aa-10	4779	4792	nsp12	LLLDKRTTCFSVAA	0.22	21
S-10	610	623	S	VLYQDVNCTEVPVA	0.22	12
S-9	816	829	S	SFIEDLLFNKVTLA	0.22	3
ORF1ab1-1400aa-10	579	592	nsp2	RLIDAMMFTSDLAT	0.23	/
ORF1ab1-1400aa-11	1176	1189	nsp3	AVFDKNLYDKLVSS	0.23	7
ORF1ab1-1400aa-12	1033	1046	nsp3	KLTDNVYIKNADIV	0.23	1
ORF1ab5783-7096aa-85	6508	6521	nsp15	ELWAKRNIKPVPEV	0.23	17
N28274-29533-4	338	351	N	KLDDKDPNFKDQVI	0.24	26
ORF1ab1-1400aa-13	383	396	nsp2	SLAEYHNESGLKTI	0.24	2
ORF1ab1-1400aa-14	903	916	nsp3	ATYYLFDESGEFKL	0.24	1
ORF1ab1387-2800aa-13	2368	2381	nsp3	IINLVQMAPISAMV	0.24	12
ORF1ab2787-4200aa-21	3122	3135	nsp4	FLAHIQWMVMFTPL	0.24	14
S-11	511	524	S	VVLSFELLHAPATV	0.24	22
ORF1ab1387-2800aa-14	1616	1629	nsp3	KTFYVLPNDDTLRV	0.25	/
ORF1ab2787-4200aa-22	3899	3912	nsp7	LLAKDTTEAFEKMV	0.25	14
ORF1ab4397-5796aa-11	4966	4979	nsp12	KLLKSIAATRGATV	0.25	16
ORF1ab1-1400aa-15	574	587	nsp2	SQYSLRLIDAMMFT	0.26	7
ORF1ab1-1400aa-16	1143	1156	nsp3	VLLAPLLSAGIFGA	0.26	2
ORF1ab1387-2800aa-15	1435	1448	nsp3	INTLNDLNETLVTM	0.26	13
ORF1ab1387-2800aa-16	1489	1502	nsp3	YLTSSSKTPEEHFI	0.26	12
ORF1ab2787-4200aa-23	4032	4045	nsp8	MLFTMLRKLDNDAL	0.26	8
ORF1ab1-1400aa-17	1378	1391	nsp3	MLAHAEETRKLMPV	0.27	7
ORF1ab1-1400aa-18	584	597	nsp2	MMFTSDLATNNLVV	0.27	2
ORF1ab1387-2800aa-17	2782	2795	nsp4	FVAAIFYLITPVHV	0.27	13
ORF1ab2787-4200aa-24	3109	3122	nsp4	YLYLTFYLTNDVSF	0.27	5
ORF1ab2787-4200aa-25	3470	3483	nsp5	WLYAAVINGDRWFL	0.27	9
ORF1ab2787-4200aa-26	3673	3686	nsp6	SLSGFKLKDCVMYA	0.27	14
ORF1ab1-1400aa-19	906	919	nsp3	YLFDESGEFKLASH	0.28	7
ORF1ab1-1400aa-20	789	802	nsp2	MLLEIKDTEKYCAL	0.28	7
ORF1ab1-1400aa-21	1242	1255	nsp3	TLEETKFLTENLLL	0.28	5
ORF1ab5783-7096aa-86	6954	6967	nsp16	FIQQKLALGGSVAI	0.28	23
ORF3a-5	72	85	ORF3a	ALSKGVHFVCNLLL	0.28	24
M-1	47	60	M	YIIKLIFLWLLWPV	0.29	26
N28274-29533-5	333	346	N	YTGAIKLDDKDPNF	0.29	26
ORF1ab1-1400aa-22	600	613	nsp2	YITGGVVQLTSQWL	0.29	1

Epitope	Start in ORF	End in ORF	Protein	Sequence	Rank	Pool
ORF1ab2787-4200aa-27	3482	3495	nsp5	FLNRFTTTLNDFNL	0.29	9
ORF1ab2787-4200aa-28	3084	3097	nsp4	LFLMSFTVLCLTPV	0.29	9
ORF1ab2787-4200aa-29	3047	3060	nsp4	IVAGGIVAIVVTCL	0.29	9
S-12	864	877	S	LLTDEMIAQYTSAL	0.29	21
ORF1ab1-1400aa-23	79	92	nsp1	APHGHVMVELVAEL	0.3	2
ORF1ab1-1400aa-24	1202	1215	nsp3	KIAEIPKEEVKPFI	0.3	7
ORF1ab1387-2800aa-18	2038	2051	nsp3	NLACEDLKPVSEEV	0.3	13
ORF1ab1387-2800aa-19	1670	1683	nsp3	ADNNCYLATALLTL	0.3	12
ORF1ab5783-7096aa-87	6477	6490	nsp15	SIINNTVYTKVDGV	0.3	23
ORF1ab5783-7096aa-88	5899	5912	nsp13	IMSDRDLYDKLQFT	0.3	17
ORF1ab2787-4200aa-30	4035	4048	nsp8	TMLRKLDNDALNNI	0.31	14
ORF1ab2787-4200aa-31	4039	4052	nsp8	KLDNDALNNIINNA	0.31	9
ORF1ab4397-5796aa-12	4854	4867	nsp12	TMCDIRQLLFVVEV	0.31	21
ORF1ab5783-7096aa-89	6690	6703	nsp15	GDFSHSQLGGLHLL	0.31	23
ORF1ab5783-7096aa-90	6241	6254	nsp14	VVKAALLADKFPVL	0.31	23
ORF1ab2787-4200aa-32	3547	3560	nsp5	SALLEDEFTPFDVV	0.32	8
ORF1ab2787-4200aa-33	4003	4016	nsp8	KMADQAMTQMYKQA	0.32	9
ORF1ab4397-5796aa-13	5465	5478	nsp13	TEETFKLSYGIATV	0.32	17
ORF1ab4397-5796aa-14	5315	5328	nsp12	AMYTPHTVLQAVGA	0.32	17
ORF1ab5783-7096aa-91	6253	6266	nsp14	VLHDIGNPKAIKCV	0.32	23
ORF1ab5783-7096aa-92	6029	6042	nsp14	NLPLQLGFSTGVNL	0.32	16
ORF3a-6	236	249	ORF3a	IVDEPEEHVQIHTI	0.32	24
ORF3a-7	67	80	ORF3a	KRWQLALSKGVHFV	0.32	24
S-13	275	288	S	FLLKYNENGTITDA	0.32	22
ORF1ab2787-4200aa-34	4168	4181	nsp9	ALAYYNTTKGGRFV	0.33	14
ORF1ab4397-5796aa-15	4993	5006	nsp12	MLKTVYSDVENPHL	0.33	16
ORF1ab5783-7096aa-93	5978	5991	nsp14	RLISMMGFKMNYQV	0.33	23
ORF3a-1	134	147	ORF3a	RSKNPLLYDANYFL	0.33	24
ORF3a-8	52	65	ORF3a	LLAVFQSASKIITL	0.33	24
M-2	56	69	M	LLWPVTLACFVLAA	0.34	26
ORF1ab4397-5796aa-16	5695	5708	nsp13	VVFDEISMATNYDL	0.34	16
ORF1ab4397-5796aa-17	5504	5517	nsp13	YVFTGYRVTKNSKV	0.34	21
ORF3a-9	135	148	ORF3a	SKNPLLYDANYFLC	0.34	24
ORF1ab1-1400aa-25	1286	1299	nsp3	IVGDVVQEGVLTAV	0.35	7
ORF1ab1-1400aa-26	641	654	nsp2	FLRDGWEIVKFIST	0.35	2
ORF1ab2787-4200aa-35	3661	3674	nsp6	MRIMTWLDMVDTSL	0.35	8
ORF1ab2787-4200aa-36	3578	3591	nsp6	GTHHWLLLTILTSL	0.35	11
ORF1ab2787-4200aa-38	3886	3899	nsp7	KLWAQCVQLHNDIL	0.35	9
ORF1ab5783-7096aa-94	6745	6758	nsp15	SVIDLLLDDFVEII	0.35	23
ORF7a-1	61	74	ORF7a	TQFAFACPDGVKHV	0.35	/
S-14	877	890	S	LLAGTITSGWTFGA	0.35	21
ORF1ab1-1400aa-27	568	581	nsp2	TILDGISQYSLRLI	0.36	7
ORF1ab1387-2800aa-20	2358	2371	nsp3	FISNSWLMWLIINL	0.36	/

Epitope	Start in ORF	End in ORF	Protein	Sequence	Rank	Pool
ORF1ab1387-2800aa-21	2600	2613	nsp3	STFNVPMEKLKTLV	0.36	12
ORF1ab1387-2800aa-22	1694	1707	nsp3	ALQDAYYRARAGEA	0.36	13
ORF1ab2787-4200aa-39	3866	3879	nsp7	KCTSVVLLSVLQQL	0.36	11
ORF1ab4397-5796aa-18	5046	5059	nsp12	RLANECAQVLSEMV	0.36	16
ORF1ab5783-7096aa-95	6881	6894	nsp16	AVLRQWLPTGTLLV	0.36	23
ORF1ab5783-7096aa-96	6744	6757	nsp15	CSVIDLLLDDFVEI	0.36	23
M-3	89	102	M	GLMWLSYFIASFRL	0.37	26
N28274-29533-6	209	222	N	RMAGNGGDAALALL	0.37	26
ORF1ab1-1400aa-28	839	852	nsp3	VNITFELDERIDKV	0.37	2
ORF1ab2787-4200aa-41	4178	4191	nsp9	GRFVLALLSDLQDL	0.37	5
ORF1ab2787-4200aa-42	3013	3026	nsp4	SLPGVFCGVDAVNL	0.37	9
ORF1ab5783-7096aa-97	6750	6763	nsp15	LLDDFVEIIKSQDL	0.37	23
ORF1ab5783-7096aa-98	6094	6107	nsp14	MLSDTLKNLSDRVV	0.37	23
ORF1ab5783-7096aa-99	5844	5857	nsp13	AVASKILGLPTQTV	0.37	23
S-15	1215	1228	S	YIWLGFIAGLIAIV	0.37	3
ORF1ab1-1400aa-29	185	198	nsp2	YVDNNFCGPDGYPL	0.38	7
ORF1ab1387-2800aa-23	2558	2571	nsp3	SVYYSQLMCQPILL	0.38	13
ORF1ab1387-2800aa-24	2265	2278	nsp3	GYREGYLNSTNVTI	0.38	12
ORF1ab2787-4200aa-43	4115	4128	nsp8	SMDNSPNLAWPLIV	0.38	11
ORF1ab4397-5796aa-1	5348	5361	nsp13	FLCCKCCYDHVIST	0.38	16
ORF1ab4397-5796aa-19	4720	4733	nsp12	PLVRKIFVDGVPFV	0.38	16
ORF3a-10	84	97	ORF3a	LLLFVTVYSHLLLV	0.38	24
S-16	983	996	S	RLDKVEAEVQIDRL	0.38	20
N28274-29533-7	394	407	N	LLPAADLDDFSKQL	0.39	26
ORF1ab1-1400aa-30	687	700	nsp2	ALCADSIIIGGAKL	0.39	1
ORF1ab1-1400aa-31	882	895	nsp3	TLQPVSELLTPLGI	0.39	1
ORF1ab1387-2800aa-25	1561	1574	nsp3	SLREVRTIKVFTTV	0.39	10
ORF1ab1387-2800aa-26	2584	2597	nsp3	AEVAVKMFDAYVNT	0.39	13
ORF1ab2787-4200aa-44	3678	3691	nsp6	KLKDCVMYASAVVL	0.39	9
ORF1ab4397-5796aa-20	5476	5489	nsp13	ATVREVLSDRELHL	0.39	21
ORF1ab4397-5796aa-21	5099	5112	nsp12	LLSTDGNKIADKYV	0.39	17
ORF1ab2787-4200aa-45	3735	3748	nsp6	ALIISVTSNYSGVV	0.4	11
ORF1ab2787-4200aa-46	2955	2968	nsp4	VLMDGSIIQFPNTY	0.4	8
ORF1ab4397-5796aa-22	5537	5550	nsp13	GTTTYKLNVGDYFV	0.4	17
ORF1ab4397-5796aa-23	5005	5018	nsp12	HLMGWDYPKCDRAM	0.4	21
ORF1ab2787-4200aa-47	3454	3467	nsp5	AQAAGTDTTITVNV	0.41	5
ORF1ab2787-4200aa-48	3160	3173	nsp4	YLKRRVVFNGVSFS	0.41	5
ORF1ab4397-5796aa-24	5722	5735	nsp13	YIGDPAQLPAPRTL	0.41	21
ORF1ab4397-5796aa-25	5575	5588	nsp13	GLYPTLNISDEFSS	0.41	15
ORF1ab5783-7096aa-100	6093	6106	nsp14	QMLSDTLKNLSDRV	0.41	23
S-17	1176	1189	S	VVNIQKEIDRLNEV	0.41	3
S-18	117	130	S	LLIVNNATNVVIKV	0.41	3
N28274-29533-8	160	173	N	QLPQGTTLPKGFYA	0.42	26

Epitope	Start in ORF	End in ORF	Protein	Sequence	Rank	Pool
ORF1ab4397-5796aa-26	5241	5254	nsp12	KTDGTLMIERFVSL	0.42	21
S-19	394	407	S	NVYADSFVIRGDEV	0.42	3
N28274-29533-9	100	113	N	KMKDLSPRWYFYYL	0.43	/
ORF1ab1-1400aa-32	47	60	nsp1	KDGTCGLVEVEKGV	0.43	7
ORF1ab1-1400aa-33	1104	1117	nsp3	VLSGHNLAKHCLHV	0.43	2
ORF1ab1-1400aa-34	463	476	nsp2	IVGDFKLNEEIAII	0.43	2
ORF1ab4187-4405aa-1	4297	4310	nsp10	MLCTHTGTGQAITV	0.43	21
ORF1ab5783-7096aa-101	6027	6040	nsp14	GTNLPLQLGFSTGV	0.43	23
ORF7a-2	95	108	ORF7a	ELYSPIFLIVAAIV	0.43	/
S-20	597	610	S	VITPGTNTSNQVAV	0.43	20
M-4	53	66	M	FLWLLWPVTLACFV	0.44	26
ORF1ab1-1400aa-35	583	596	nsp2	AMMFTSDLATNNLV	0.44	7
ORF1ab1387-2800aa-27	1796	1809	nsp3	YLVQQESPFVMMSA	0.44	13
ORF1ab2787-4200aa-49	3804	3817	nsp6	FRLTLGVYDYLVST	0.44	8
ORF3a-11	40	53	ORF3a	SLPFGWLIVGVALL	0.44	24
ORF7a-3	90	103	ORF7a	QEEVQELYSPIFLI	0.44	/
S-21	805	818	S	ILPDPSKPSKRSFI	0.44	3
M-5	69	82	M	AVYRINWITGGIAI	0.45	26
M-6	50	63	M	KLIFLWLLWPVTLA	0.45	26
ORF1ab1-1400aa-36	851	864	nsp3	KVLNEKCSAYTVEL	0.45	2
ORF1ab1-1400aa-37	697	710	nsp2	GAKLKALNLGETFV	0.45	7
ORF1ab1387-2800aa-28	2328	2341	nsp3	FLAYILFTRFFYVL	0.45	10
ORF1ab4397-5796aa-27	5360	5373	nsp13	STSHKLVLSVNPYV	0.45	21
ORF1ab5783-7096aa-102	6628	6641	nsp15	FNYYKKVDGVVQQL	0.45	23
ORF1ab5783-7096aa-103	6240	6253	nsp14	MVVKAALLADKFPV	0.45	17
ORF7a-4	43	56	ORF7a	NSPFHPLADNKFAL	0.45	/
M-7	103	116	M	FARTRSMWSFNPET	0.46	26
ORF1ab1-1400aa-38	26	39	nsp1	VLVRGFGDSVEEVL	0.46	1
ORF1ab1387-2800aa-29	1682	1695	nsp3	TLQQIELKFNPPAL	0.46	13
ORF1ab2787-4200aa-50	3103	3116	nsp4	GVYSVIYLYLTFYL	0.46	8
ORF1ab2787-4200aa-51	3130	3143	nsp4	VMFTPLVPFWITIA	0.46	8
ORF1ab1-1400aa-39	546	559	nsp2	SIFSRTLETAQNSV	0.47	7
ORF1ab1-1400aa-40	1358	1371	nsp3	SIISNEKQEILGTV	0.47	2
ORF1ab1-1400aa-41	95	108	nsp1	IQYGRSGETLGVLV	0.47	7
ORF1ab1387-2800aa-30	2372	2385	nsp3	VQMAPISAMVRMYI	0.47	10
ORF1ab1387-2800aa-31	2327	2340	nsp3	WFLAYILFTRFFYV	0.47	10
ORF1ab1387-2800aa-32	2589	2602	nsp3	KMFDAYVNTFSSTF	0.47	10
ORF1ab2787-4200aa-52	2960	2973	nsp4	SIIQFPNTYLEGSV	0.47	8
ORF1ab4397-5796aa-28	4905	4918	nsp12	RLYYDSMSYEDQDA	0.47	16
ORF1ab5783-7096aa-104	6572	6585	nsp15	TVFFDGRVDGQVDL	0.47	23
ORF6-2	31	44	ORF6	YIINLIIKNLSKSL	0.47	25
ORF8-1	103	116	ORF8	SFYEDFLEYHDVRV	0.47	/
E-2	45	58	E	NIVNVSLVKPSFYV	0.48	25

Epitope	Start in ORF	End in ORF	Protein	Sequence	Rank	Pool
ORF1ab1387-2800aa-33	2571	2584	nsp3	LLDQALVSDVGDSA	0.48	10
ORF1ab1387-2800aa-34	2217	2230	nsp3	YLKSPNFSKLINII	0.48	10
ORF1ab1387-2800aa-35	1545	1558	nsp3	HLDGEVITFDNLKT	0.48	10
ORF1ab2787-4200aa-53	3635	3648	nsp6	FLCLFLLPSLATVA	0.48	6
ORF1ab4397-5796aa-29	5208	5221	nsp12	HTMLVKQGDDYVYL	0.48	18
ORF7b-2	21	34	ORF7b	VLIMLIIFWFSLEL	0.48	/
S-22	991	1004	S	VQIDRLITGRLQSL	0.48	21
ORF1ab1-1400aa-42	1109	1122	nsp3	NLAKHCLHVVGPNV	0.49	7
ORF1ab1387-2800aa-36	2126	2139	nsp3	GLAAVNSVPWDTIA	0.49	10
ORF1ab2787-4200aa-54	3011	3024	nsp4	YRSLPGVFCGVDAV	0.49	5
ORF1ab2787-4200aa-55	3727	3740	nsp6	LDQAISMWALIISV	0.49	11
ORF1ab4397-5796aa-30	5041	5054	nsp12	SHRFYRLANECAQV	0.49	15
ORF1ab5783-7096aa-105	6448	6461	nsp15	FTRLQSLENVAFNV	0.49	/
ORF1ab1387-2800aa-37	2490	2503	nsp3	IVDSVTVKNGSIHL	0.5	13
ORF1ab1387-2800aa-38	2332	2345	nsp3	ILFTRFFYVLGLAA	0.5	10
ORF1ab2787-4200aa-1	3827	3840	nsp6	GLLPPKNSIDAFKL	0.5	8
ORF1ab2787-4200aa-56	3898	3911	nsp7	ILLAKDTTEAFEKM	0.5	9
ORF1ab2787-4200aa-57	3117	3130	nsp4	TNDVSFLAHIQWMV	0.5	11
ORF1ab2787-4200aa-58	3795	3808	nsp6	GLFCLLNRYFRLTL	0.5	6
ORF1ab5783-7096aa-106	6892	6905	nsp16	LLVDSDLNDFVSDA	0.5	17
S-23	55	68	S	FLPFFSNVTWFHAI	0.5	3
M-8	127	140	M	TILTRPLLESELVI	0.51	26
M-9	15	28	M	KLLEQWNLVIGFLF	0.51	26
ORF1ab1-1400aa-43	82	95	nsp1	GHVMVELVAELEGI	0.51	1
ORF1ab2787-4200aa-59	3666	3679	nsp6	WLDMVDTSLSGFKL	0.51	5
ORF1ab2787-4200aa-60	4109	4122	nsp8	VQLSEISMDNSPNL	0.51	11
ORF1ab4397-5796aa-31	4673	4686	nsp12	KLFDRYFKYWDQTY	0.51	18
ORF1ab5783-7096aa-107	7039	7052	nsp16	SYSLFDMSKFPLKL	0.51	16
ORF8-2	68	81	ORF8	KSPIQYIDIGNYTV	0.51	25
S-24	77	90	S	KRFDNPVLPFNDGV	0.51	22
ORF1ab1-1400aa-44	1355	1368	nsp3	ILPSIISNEKQEIL	0.52	7
ORF1ab1387-2800aa-39	2342	2355	nsp3	GLAAIMQLFFSYFA	0.52	13
ORF1ab1387-2800aa-40	2629	2642	nsp3	VLSTFISAARQGFV	0.52	13
ORF1ab5783-7096aa-108	6089	6102	nsp14	IKIVQMLSDTLKNL	0.52	15
ORF3a-12	139	152	ORF3a	LLYDANYFLCWHTN	0.52	24
S-25	158	171	S	RVYSSANNCTFEYV	0.52	22
S-26	821	834	S	LLFNKVTLADAGFI	0.52	20
ORF1ab2787-4200aa-61	3720	3733	nsp6	KVYYGNALDQAISM	0.53	11
ORF1ab2787-4200aa-62	2872	2885	nsp4	TILRTTNGDFLHFL	0.53	9
ORF7a-5	4	17	ORF7a	ILFLALITLATCEL	0.53	25
ORF1ab2787-4200aa-63	2864	2877	nsp4	FVVPGLPGTILRTT	0.54	8
E-3	20	33	E	FLAFVVFLLVTLAI	0.55	25
ORF1ab1387-2800aa-41	2591	2604	nsp3	FDAYVNTFSSTFNV	0.55	10

Epitope	Start in ORF	End in ORF	Protein	Sequence	Rank	Pool
ORF1ab1387-2800aa-42	2380	2393	nsp3	MVRMYIFFASFYYV	0.55	10
ORF1ab4397-5796aa-32	5690	5703	nsp13	TTADIVVFDEISMA	0.55	18
ORF1ab5783-7096aa-109	6613	6626	nsp15	SLNGVTLIGEAVKT	0.55	/
ORF1ab5783-7096aa-110	6032	6045	nsp14	LQLGFSTGVNLVAV	0.55	/
ORF10-1	5	18	ORF10	NVFAFPFTIYSLLL	0.56	25
ORF1ab1387-2800aa-43	2270	2283	nsp3	YLNSTNVTIATYCT	0.56	10
ORF1ab2787-4200aa-64	3793	3806	nsp6	YFGLFCLLNRYFRL	0.56	6
ORF1ab2787-4200aa-65	3950	3963	nsp8	SLPSYAAFATAQEA	0.56	11
ORF1ab2787-4200aa-66	3484	3497	nsp5	NRFTTTLNDFNLVA	0.56	8
ORF1ab2787-4200aa-67	3165	3178	nsp4	VVFNGVSFSTFEEA	0.56	11
ORF1ab2787-4200aa-68	3737	3750	nsp6	IISVTSNYSGVVTT	0.56	11
ORF1ab4397-5796aa-33	5615	5628	nsp13	FAIGLALYYPSARI	0.56	15
ORF1ab4397-5796aa-34	4780	4793	nsp12	LLDKRTTCFSVAAL	0.56	18
ORF1ab5783-7096aa-111	5926	5939	nsp14	AENVTGLFKDCSKV	0.56	20
ORF3a-13	105	118	ORF3a	FLYLYALVYFLQSI	0.56	/
ORF3a-14	28	41	ORF3a	FVRATATIPIQASL	0.56	24
S-27	331	344	S	NITNLCPFGEVFNA	0.56	3
S-28	921	934	S	KLIANQFNSAIGKI	0.56	22
E-4	26	39	E	FLLVTLAILTALRL	0.57	25
M-10	107	120	M	RSMWSFNPETNILL	0.57	26
ORF1ab1387-2800aa-1	2575	2588	nsp3	ALVSDVGDSAEVAV	0.57	/
ORF1ab1387-2800aa-44	2193	2206	nsp3	SMPTTIAKNTVKSV	0.57	10
ORF1ab1387-2800aa-45	2720	2733	nsp3	SLSEQLRKQIRSAA	0.57	10
ORF1ab2787-4200aa-69	3908	3921	nsp7	FEKMVSLLSVLLSM	0.57	5
ORF1ab2787-4200aa-70	3320	3333	nsp5	LLIRKSNHNFLVQA	0.57	6
ORF1ab4397-5796aa-35	4626	4639	nsp12	VDSYYSLLMPILTL	0.57	18
ORF1ab5783-7096aa-112	6687	6700	nsp15	IVYGDFSHSQLGGL	0.57	17
ORF3a-15	212	225	ORF3a	YQLYSTQLSTDTGV	0.57	/
ORF3a-16	37	50	ORF3a	IQASLPFGWLIVGV	0.57	24
S-29	910	923	S	GVTQNVLYENQKLI	0.57	22
ORF1ab2787-4200aa-71	3207	3220	nsp4	YLALYNKYKYFSGA	0.58	11
ORF1ab4397-5796aa-36	5751	5764	nsp13	RLMKTIGPDMFLGT	0.58	16
ORF1ab4397-5796aa-37	4636	4649	nsp12	ILTLTRALTAESHV	0.58	18
ORF1ab5783-7096aa-113	7009	7022	nsp16	YLGKPREQIDGYVM	0.58	/
S-30	1132	1145	S	IVNNTVYDPLQPEL	0.58	12
ORF1ab1-1400aa-45	615	628	nsp2	NIFGTVYEKLKPVL	0.59	7
ORF1ab2787-4200aa-72	3753	3766	nsp6	FLARGIVFMCVEYC	0.59	11
ORF1ab2787-4200aa-73	3843	3856	nsp6	KLLGVGGKPCIKVA	0.59	11
ORF3a-17	95	108	ORF3a	LLVAAGLEAPFLYL	0.59	24
E-5	38	51	E	RLCAYCCNIVNVSL	0.6	25
ORF1ab1-1400aa-46	901	914	nsp3	SMATYYLFDESGEF	0.6	7
ORF1ab1387-2800aa-46	1770	1783	nsp3	YMGTLSYEQFKKGV	0.6	10
S-31	1185	1198	S	RLNEVAKNLNESLI	0.6	20

Epitope	Start in ORF	End in ORF	Protein	Sequence	Rank	Pool
M-11	108	121	M	SMWSFNPETNILLN	0.61	/
M-12	84	97	M	MACLVGLMWLSYFI	0.61	/
ORF1ab2787-4200aa-74	3004	3017	nsp4	WVLNNDYYRSLPGV	0.61	11
ORF1ab2787-4200aa-75	4110	4123	nsp8	QLSEISMDNSPNLA	0.61	11
ORF1ab2787-4200aa-76	4094	4107	nsp8	ALWEIQQVVDADSK	0.61	11
ORF1ab5783-7096aa-115	6104	6117	nsp14	DRVVFVLWAHGFEL	0.61	/
ORF1ab5783-7096aa-116	5823	5836	nsp13	FLTRNPAWRKAVFI	0.61	16
E-6	11	24	E	TLIVNSVLLFLAFV	0.62	25
ORF1ab1-1400aa-47	209	222	nsp2	GKASCTLSEQLDFI	0.62	1
ORF1ab1387-2800aa-47	1444	1457	nsp3	TLVTMPLGYVTHGL	0.62	10
ORF1ab4397-5796aa-38	5763	5776	nsp13	GTCRRCPAEIVDTV	0.62	18
ORF1ab4397-5796aa-39	5603	5616	nsp13	TLQGPPGTGKSHFA	0.62	15
ORF1ab5783-7096aa-117	7081	7094	nsp16	RENNRVVISSDVLV	0.62	17
ORF1ab5783-7096aa-118	6420	6433	nsp14	LDAYNMMISAGFSL	0.62	20
S-32	692	705	S	IIAYTMSLGAENSV	0.62	3
S-33	247	260	S	SYLTPGDSSSGWTA	0.62	20
N28274-29533-10	381	394	N	ALPQRQKKQQTVTL	0.63	26
ORF1ab1-1400aa-48	527	540	nsp2	KSILSPLYAFASEA	0.63	7
ORF1ab1-1400aa-49	15	28	nsp1	QLSLPVLQVRDVLV	0.63	2
ORF1ab1387-2800aa-48	2071	2084	nsp3	VVGDIILKPANNSL	0.63	13
ORF1ab1387-2800aa-49	1895	1908	nsp3	KLDNYYKKDNSYFT	0.63	10
ORF1ab2787-4200aa-77	3582	3595	nsp6	WLLLTILTSLLVLV	0.63	8
ORF1ab2787-4200aa-78	3028	3041	nsp4	TNMFTPLIQPIGAL	0.63	6
ORF1ab4187-4405aa-2	4278	4291	nsp10	KAYKDYLASGGQPI	0.63	21
ORF7b-3	4	17	ORF7b	LSLIDFYLCFLAFL	0.63	24
ORF1ab2787-4200aa-2	3292	3305	nsp5	GLWLDDVVYCPRHV	0.64	5
ORF1ab2787-4200aa-79	3603	3616	nsp6	FFFLYENAFLPFAM	0.64	11
ORF1ab2787-4200aa-80	3593	3606	nsp6	VLVQSTQWSLFFFL	0.64	11
ORF1ab2787-4200aa-81	3094	3107	nsp4	LTPVYSFLPGVYSV	0.64	6
ORF1ab4397-5796aa-40	5739	5752	nsp13	GTLEPEYFNSVCRL	0.64	18
ORF1ab5783-7096aa-119	6493	6506	nsp15	ELFENKTTLPVNVA	0.64	/
ORF3a-18	107	120	ORF3a	YLYALVYFLQSINF	0.64	/
S-34	104	117	S	WIFGTTLDSKTQSL	0.64	20
ORF1ab1-1400aa-50	1226	1239	nsp3	KQDDKKIKACVEEV	0.65	4
ORF1ab1387-2800aa-50	1425	1438	nsp3	YTSKTTVASLINTL	0.65	10
ORF1ab1387-2800aa-51	2233	2246	nsp3	FLLLSVCLGSLIYS	0.65	10
ORF1ab1387-2800aa-52	2541	2554	nsp3	IVFDGKSKCEESSA	0.65	12
ORF1ab1387-2800aa-53	1689	1702	nsp3	KFNPPALQDAYYRA	0.65	/
ORF1ab2787-4200aa-82	3888	3901	nsp7	WAQCVQLHNDILLA	0.65	5
ORF1ab2787-4200aa-83	3339	3352	nsp5	RVIGHSMQNCVLKL	0.65	5
ORF1ab2787-4200aa-84	2942	2955	nsp4	SVAYESLRPDTRYV	0.65	11
S-35	741	754	S	YICGDSTECSNLLL	0.65	22
ORF1ab1387-2800aa-54	2783	2796	nsp4	VAAIFYLITPVHVM	0.66	12

Epitope	Start in ORF	End in ORF	Protein	Sequence	Rank	Pool
ORF1ab1387-2800aa-55	2285	2298	nsp3	SIPCSVCLSGLDSL	0.66	12
ORF1ab2787-4200aa-85	4060	4073	nsp8	NIIPLTTAAKLMVV	0.66	19
ORF1ab4397-5796aa-41	5681	5694	nsp13	FCTVNALPETTADI	0.66	15
S-36	1055	1068	S	SAPHGVVFLHVTYV	0.66	22
ORF1ab1-1400aa-51	801	814	nsp2	ALAPNMMVTNNTFT	0.67	4
ORF1ab1-1400aa-52	889	902	nsp3	LLTPLGIDLDEWSM	0.67	4
ORF1ab1387-2800aa-56	2564	2577	nsp3	LMCQPILLLDQALV	0.67	/
ORF1ab2787-4200aa-86	3078	3091	nsp4	VAFNTLLFLMSFTV	0.67	6
ORF1ab2787-4200aa-87	3025	3038	nsp4	NLLTNMFTPLIQPI	0.67	19
ORF1ab4397-5796aa-42	5766	5779	nsp13	RRCPAEIVDTVSAL	0.67	15
ORF1ab4397-5796aa-43	5106	5119	nsp12	KIADKYVRNLQHRL	0.67	17
ORF1ab4397-5796aa-44	4732	4745	nsp12	FVVSTGYHFRELGV	0.67	20
ORF1ab5783-7096aa-120	6704	6717	nsp15	IGLAKRFKESPFEL	0.67	17
N28274-29533-11	233	246	N	KMSGKGQQQQGQTV	0.68	26
ORF1ab1-1400aa-53	595	608	nsp2	LVVMAYITGGVVQL	0.68	1
ORF1ab1-1400aa-54	528	541	nsp2	SILSPLYAFASEAA	0.68	4
ORF1ab1387-2800aa-57	2611	2624	nsp3	TLVATAEAELAKNV	0.68	12
ORF1ab2787-4200aa-88	3834	3847	nsp6	SIDAFKLNIKLLGV	0.68	5
ORF1ab5783-7096aa-121	6594	6607	nsp15	LITEGSVKGLQPSV	0.68	/
ORF1ab5783-7096aa-122	6438	6451	nsp14	QFDTYNLWNTFTRL	0.68	17
E-7	6	19	E	SEETGTLIVNSVLL	0.69	25
ORF1ab1-1400aa-55	662	675	nsp2	GQIVTCAKEIKESV	0.69	4
ORF1ab1387-2800aa-58	1984	1997	nsp3	KLLHKPIVWHVNNA	0.69	/
ORF1ab4397-5796aa-45	4789	4802	nsp12	SVAALTNNVAFQTV	0.69	16
ORF1ab5783-7096aa-123	6980	6993	nsp16	KLMGHFAWWTAFVT	0.69	/
ORF1ab5783-7096aa-124	6924	6937	nsp16	LIISDMYDPKTKNV	0.69	/
E-8	57	70	E	YVYSRVKNLNSSRV	0.7	25
ORF1ab1-1400aa-56	734	747	nsp2	KAPKEIIFLEGETL	0.7	4
ORF1ab1-1400aa-57	1174	1187	nsp3	YLAVFDKNLYDKLV	0.7	4
ORF1ab1387-2800aa-59	1428	1441	nsp3	KTTVASLINTLNDL	0.7	12
ORF1ab4397-5796aa-46	5143	5156	nsp12	KHFSMMILSDDAVV	0.7	18
ORF1ab5783-7096aa-125	5919	5932	nsp13	RNVATLQAENVTGL	0.7	15
ORF3a-19	102	115	ORF3a	EAPFLYLYALVYFL	0.7	24
ORF3a-20	93	106	ORF3a	HLLLVAAGLEAPFL	0.7	24
ORF7b-4	28	41	ORF7b	FWFSLELQDHNETC	0.7	25
S-37	264	277	S	AYYVGYLQPRTFLL	0.7	3
M-13	10	23	M	VEELKKLLEQWNLV	0.71	26
ORF1ab1387-2800aa-60	2563	2576	nsp3	QLMCQPILLLDQAL	0.71	12
ORF1ab2787-4200aa-3	3605	3618	nsp6	FLYENAFLPFAMGI	0.71	5
ORF1ab2787-4200aa-89	3546	3559	nsp5	GSALLEDEFTPFDV	0.71	9
ORF1ab2787-4200aa-90	3748	3761	nsp6	VTTVMFLARGIVFM	0.71	9
ORF1ab2787-4200aa-91	3129	3142	nsp4	MVMFTPLVPFWITI	0.71	19
ORF1ab2787-4200aa-92	3654	3667	nsp6	YMPASWVMRIMTWL	0.71	8

Epitope	Start in ORF	End in ORF	Protein	Sequence	Rank	Pool
ORF1ab4397-5796aa-47	4699	4712	nsp12	ILHCANFNVLFSTV	0.71	15
ORF1ab5783-7096aa-126	6535	6548	nsp15	TVIWDYKRDAPAHI	0.71	/
S-39	881	894	S	TITSGWTFGAGAAL	0.71	22
ORF1ab1387-2800aa-61	2297	2310	nsp3	SLDTYPSLETIQIT	0.72	13
ORF1ab2787-4200aa-93	3699	3712	nsp6	TVYDDGARRVWTLM	0.72	19
ORF1ab4397-5796aa-48	5679	5692	nsp13	YVFCTVNALPETTA	0.72	20
ORF1ab4397-5796aa-49	5223	5236	nsp12	YPDPSRILGAGCFV	0.72	15
ORF1ab4397-5796aa-50	4766	4779	nsp12	YAADPAMHAASGNL	0.72	15
ORF1ab4397-5796aa-51	4410	4423	nsp12	RLTPCGTGTSTDVV	0.72	16
ORF1ab5783-7096aa-127	5930	5943	nsp14	TGLFKDCSKVITGL	0.72	16
S-40	978	991	S	NDILSRLDKVEAEV	0.72	3
S-41	1050	1063	S	MSFPQSAPHGVVFL	0.72	3
ORF10-2	20	33	ORF10	RMNSRNYIAQVDVV	0.73	/
ORF1ab2787-4200aa-94	3364	3377	nsp5	YKFVRIQPGQTFSV	0.73	9
S-42	1109	1122	S	FYEPQIITTDNTFV	0.73	22
ORF1ab1-1400aa-58	524	537	nsp2	GEQKSILSPLYAFA	0.74	4
ORF1ab1387-2800aa-62	1464	1477	nsp3	RYMRSLKVPATVSV	0.74	12
ORF1ab1387-2800aa-63	2776	2789	nsp4	VTLVFLFVAAIFYL	0.74	/
ORF1ab2787-4200aa-95	3188	3201	nsp4	EMYLKLRSDVLLPL	0.74	5
ORF1ab4397-5796aa-52	5455	5468	nsp13	KLFAAETLKATEET	0.74	17
S-43	686	699	S	SVASQSIIAYTMSL	0.74	22
S-44	848	861	S	DLICAQKFNGLTVL	0.74	3
S-45	563	576	S	QQFGRDIADTTDAV	0.74	3
ORF1ab1387-2800aa-64	2425	2438	nsp3	TIVNGVRRSFYVYA	0.75	12
ORF1ab2787-4200aa-96	3237	3250	nsp4	ALNDFSNSGSDVLY	0.75	19
ORF1ab5783-7096aa-129	6846	6859	nsp16	TQLCQYLNTLTLAV	0.75	/
ORF6-3	23	36	ORF6	KVSIWNLDYIINLI	0.75	25
ORF7a-6	11	24	ORF7a	TLATCELYHYQECV	0.75	/
S-1	220	233	S	FSALEPLVDLPIGI	0.75	3
N28274-29533-1	120	133	N	GLPYGANKDGIIWV	0.76	26
ORF1ab2787-4200aa-4	3634	3647	nsp6	AFLCLFLLPSLATV	0.76	6
ORF1ab2787-4200aa-97	3099	3112	nsp4	SFLPGVYSVIYLYL	0.76	19
ORF1ab2787-4200aa-98	3602	3615	nsp6	LFFFLYENAFLPFA	0.76	5
ORF1ab4187-4405aa-3	4233	4246	nsp9	GLNNLNRGMVLGSL	0.76	9
ORF1ab4397-5796aa-53	5749	5762	nsp13	VCRLMKTIGPDMFL	0.76	18
ORF1ab4397-5796aa-54	4631	4644	nsp12	SLLMPILTLTRALT	0.76	20
ORF1ab4397-5796aa-55	4557	4570	nsp12	FVENPDILRVYANL	0.76	16
ORF6-1	3	16	ORF6	HLVDFQVTIAEILL	0.76	26
ORF7a-7	89	102	ORF7a	RQEEVQELYSPIFL	0.76	25
ORF7b-5	13	26	ORF7b	FLAFLLFLVLIMLI	0.76	/
ORF1ab1-1400aa-59	103	116	nsp1	TLGVLVPHVGEIPV	0.77	4
ORF1ab1387-2800aa-65	1540	1553	nsp3	NPTTFHLDGEVITF	0.77	/
ORF1ab1387-2800aa-66	2085	2098	nsp3	KITEEVGHTDLMAA	0.77	12

Epitope	Start in ORF	End in ORF	Protein	Sequence	Rank	Pool
ORF1ab2787-4200aa-100	3100	3113	nsp4	FLPGVYSVIYLYLT	0.77	19
ORF1ab2787-4200aa-99	3110	3123	nsp4	LYLTFYLTNDVSFL	0.77	19
ORF1ab5783-7096aa-130	6870	6883	nsp16	AGSDKGVAPGTAVL	0.77	/
ORF1ab5783-7096aa-131	6414	6427	nsp14	NEYRLYLDAYNMMI	0.77	16
S-46	1119	1132	S	NTFVSGNCDVVIGI	0.77	20
N28274-29533-12	311	324	N	ASAFFGMSRIGMEV	0.78	26
ORF1ab1-1400aa-60	1382	1395	nsp3	AEETRKLMPVCVET	0.78	1
ORF1ab5783-7096aa-132	6749	6762	nsp15	LLLDDFVEIIKSQD	0.78	17
ORF3a-21	90	103	ORF3a	VYSHLLLVAAGLEA	0.78	24
S-47	497	510	S	FQPTNGVGYQPYRV	0.78	22
ORF1ab1-1400aa-61	110	123	nsp1	HVGEIPVAYRKVLL	0.79	4
ORF1ab1387-2800aa-67	2237	2250	nsp3	SVCLGSLIYSTAAL	0.79	12
ORF1ab1387-2800aa-68	1761	1774	nsp3	TLKGVEAVMYMGTL	0.79	13
ORF1ab1387-2800aa-69	1456	1469	nsp3	GLNLEEAARYMRSL	0.79	12
ORF1ab2787-4200aa-101	2856	2869	nsp4	AVITREVGFVVPGL	0.79	8
ORF1ab2787-4200aa-102	3421	3434	nsp5	SFCYMHHMELPTGV	0.79	5
ORF1ab4397-5796aa-56	5570	5583	nsp13	YVRITGLYPTLNIS	0.79	16
S-48	1103	1116	S	FVTQRNFYEPQIIT	0.79	3
ORF1ab1-1400aa-62	40	53	nsp1	SEARQHLKDGTCGL	0.8	2
ORF1ab1-1400aa-63	359	372	nsp2	YLPQNAVVKIYCPA	0.8	2
ORF1ab2787-4200aa-103	3894	3907	nsp7	LHNDILLAKDTTEA	0.8	19
ORF3a-22	2	15	ORF3a	DLFMRIFTIGTVTL	0.8	24
E-9	18	31	E	LLFLAFVVFLLVTL	0.81	25
ORF1ab1-1400aa-64	51	64	nsp1	CGLVEVEKGVLPQL	0.81	1
ORF1ab1-1400aa-65	475	488	nsp2	IILASFSASTSAFV	0.81	4
ORF1ab1-1400aa-66	1387	1400	nsp3	KLMPVCVETKAIVS	0.82	2
ORF1ab1-1400aa-67	101	114	nsp1	GETLGVLVPHVGEI	0.82	1
ORF1ab1387-2800aa-70	1440	1453	nsp3	DLNETLVTMPLGYV	0.82	/
ORF1ab1387-2800aa-72	2343	2356	nsp3	LAAIMQLFFSYFAV	0.82	12
S-49	871	884	S	AQYTSALLAGTITS	0.82	22
ORF1ab1-1400aa-1	729	742	nsp2	LLMPLKAPKEIIFL	0.83	2
ORF1ab1-1400aa-68	25	38	nsp1	DVLVRGFGDSVEEV	0.83	4
ORF1ab1387-2800aa-73	2145	2158	nsp3	FLNKVVSTTTNIVT	0.83	13
ORF1ab1387-2800aa-74	1712	1725	nsp3	ALILAYCNKTVGEL	0.83	14
ORF1ab2787-4200aa-104	3088	3101	nsp4	SFTVLCLTPVYSFL	0.83	6
S-50	898	911	S	FAMQMAYRFNGIGV	0.83	3
ORF1ab1-1400aa-69	1270	1283	nsp3	LVSDIDITFLKKDA	0.84	1
ORF1ab1-1400aa-70	1367	1380	nsp3	ILGTVSWNLREMLA	0.84	1
ORF1ab1-1400aa-71	739	752	nsp2	IIFLEGETLPTEVL	0.84	4
ORF1ab1-1400aa-72	1299	1312	nsp3	VVIPTKKAGGTTEM	0.84	2
ORF1ab1387-2800aa-75	2291	2304	nsp3	CLSGLDSLDTYPSL	0.84	14
ORF1ab2787-4200aa-105	4183	4196	nsp9	ALLSDLQDLKWARF	0.84	19
ORF1ab2787-4200aa-106	3518	3531	nsp5	AQTGIAVLDMCASL	0.84	6

Epitope	Start in ORF	End in ORF	Protein	Sequence	Rank	Pool
ORF1ab2787-4200aa-107	3511	3524	nsp5	DILGPLSAQTGIAV	0.84	19
ORF1ab4187-4405aa-4	4247	4260	nsp10	AATVRLQAGNATEV	0.84	8
ORF1ab4397-5796aa-57	5274	5287	nsp12	HLYLQYIRKLHDEL	0.84	15
ORF1ab4397-5796aa-58	4921	4934	nsp12	AYTKRNVIPTITQM	0.84	20
ORF1ab5783-7096aa-134	6968	6981	nsp16	KITEHSWNADLYKL	0.84	/
ORF1ab5783-7096aa-135	6465	6478	nsp15	GHFDGQQGEVPVSI	0.84	20
ORF1ab5783-7096aa-136	6109	6122	nsp14	VLWAHGFELTSMKY	0.84	/
ORF3a-2	231	244	ORF3a	FIYNKIVDEPEEHV	0.84	24
S-51	23	36	S	QLPPAYTNSFTRGV	0.84	3
ORF1ab1-1400aa-2	3	16	nsp1	SLVPGFNEKTHVQL	0.85	1
ORF1ab1-1400aa-73	578	591	nsp2	LRLIDAMMFTSDLA	0.85	2
ORF1ab1387-2800aa-76	2083	2096	nsp3	SLKITEEVGHTDLM	0.85	14
ORF1ab2787-4200aa-108	3640	3653	nsp6	LLPSLATVAYFNMV	0.85	8
ORF1ab2787-4200aa-109	3881	3894	nsp7	VESSSKLWAQCVQL	0.85	19
ORF1ab4397-5796aa-59	5317	5330	nsp12	YTPHTVLQAVGACV	0.85	18
ORF1ab4397-5796aa-60	4939	4952	nsp12	AISAKNRARTVAGV	0.85	18
ORF1ab4397-5796aa-61	4724	4737	nsp12	KIFVDGVPFVVSTG	0.85	18
ORF1ab4397-5796aa-62	4721	4734	nsp12	LVRKIFVDGVPFVV	0.85	18
ORF1ab5783-7096aa-137	6488	6501	nsp15	DGVDVELFENKTTL	0.85	15
ORF7a-8	83	96	ORF7a	SPKLFIRQEEVQEL	0.85	25
S-52	713	726	S	AIPTNFTISVTTEI	0.85	13
ORF1ab1-1400aa-74	894	907	nsp3	GIDLDEWSMATYYL	0.86	4
ORF1ab1-1400aa-75	728	741	nsp2	GLLMPLKAPKEIIF	0.86	4
ORF1ab1387-2800aa-77	1465	1478	nsp3	YMRSLKVPATVSVS	0.86	14
ORF1ab4397-5796aa-2	5054	5067	nsp12	VLSEMVMCGGSLYV	0.86	21
S-55	852	865	S	AQKFNGLTVLPPLL	0.86	22
M-14	55	68	M	WLLWPVTLACFVLA	0.87	25
ORF1ab1387-2800aa-78	2322	2335	nsp3	GLVAEWFLAYILFT	0.87	14
ORF1ab2787-4200aa-110	2853	2866	nsp4	LIAAVITREVGFVV	0.87	6
ORF1ab2787-4200aa-111	3632	3645	nsp6	KHAFLCLFLLPSLA	0.87	19
ORF1ab4187-4405aa-5	4282	4295	nsp10	DYLASGGQPITNCV	0.87	/
ORF3a-23	136	149	ORF3a	KNPLLYDANYFLCW	0.87	24
ORF1ab1387-2800aa-3	2225	2238	nsp3	KLINIIIWFLLLSV	0.88	/
ORF1ab2787-4200aa-112	3928	3941	nsp7	NKLCEEMLDNRATL	0.88	19
ORF1ab4397-5796aa-63	5671	5684	nsp13	KVNSTLEQYVFCTV	0.88	18
ORF1ab5783-7096aa-138	6481	6494	nsp15	NTVYTKVDGVDVEL	0.88	/
ORF1ab5783-7096aa-140	5839	5852	nsp13	YNSQNAVASKILGL	0.88	/
ORF1ab1-1400aa-76	98	111	nsp1	GRSGETLGVLVPHV	0.89	1
ORF1ab1387-2800aa-79	2511	2524	nsp3	KTYERHSLSHFVNL	0.89	14
ORF1ab4397-5796aa-64	5149	5162	nsp12	ILSDDAVVCFNSTY	0.89	17
ORF1ab4397-5796aa-65	4456	4469	nsp12	NLIDSYFVVKRHTF	0.89	20
ORF1ab5783-7096aa-141	7011	7024	nsp16	GKPREQIDGYVMHA	0.89	16
S-57	1128	1141	S	VVIGIVNNTVYDPL	0.89	22

Epitope	Start in ORF	End in ORF	Protein	Sequence	Rank	Pool
ORF1ab1-1400aa-77	52	65	nsp1	GLVEVEKGVLPQLE	0.9	4
ORF1ab1387-2800aa-80	1591	1604	nsp3	GQQFGPTYLDGADV	0.9	13
ORF1ab2787-4200aa-113	4036	4049	nsp8	MLRKLDNDALNNII	0.9	19
ORF1ab2787-4200aa-114	3710	3723	nsp6	TLMNVLTLVYKVYY	0.9	19
ORF1ab2787-4200aa-115	3861	3874	nsp7	KMSDVKCTSVVLLS	0.9	5
ORF1ab2787-4200aa-116	3698	3711	nsp6	RTVYDDGARRVWTL	0.9	19
ORF1ab4397-5796aa-66	5142	5155	nsp12	RKHFSMMILSDDAV	0.9	18
ORF3a-24	215	228	ORF3a	YSTQLSTDTGVEHV	0.9	/
ORF7b-1	5	18	ORF7b	SLIDFYLCFLAFLL	0.9	/
S-59	1003	1016	S	SLQTYVTQQLIRAA	0.9	22
ORF1ab1-1400aa-78	636	649	nsp2	KEGVEFLRDGWEIV	0.91	4
ORF1ab1-1400aa-79	1230	1243	nsp3	KKIKACVEEVTTTL	0.91	4
ORF1ab1387-2800aa-81	1537	1550	nsp3	YTSNPTTFHLDGEV	0.91	/
ORF1ab2787-4200aa-117	4098	4111	nsp8	IQQVVDADSKIVQL	0.91	6
ORF1ab2787-4200aa-118	3183	3196	nsp4	FLLNKEMYLKLRSD	0.91	19
ORF1ab4397-5796aa-67	5018	5031	nsp12	MPNMLRIMASLVLA	0.91	15
ORF1ab4397-5796aa-68	4926	4939	nsp12	NVIPTITQMNLKYA	0.91	18
ORF1ab4397-5796aa-69	4851	4864	nsp12	NLPTMCDIRQLLFV	0.91	18
S-60	622	635	S	VAIHADQLTPTWRV	0.91	/
ORF1ab1-1400aa-80	1133	1146	nsp3	SAYENFNQHEVLLA	0.92	4
ORF1ab1-1400aa-81	955	968	nsp3	YQGKPLEFGATSAA	0.92	1
ORF1ab1387-2800aa-4	1432	1445	nsp3	ASLINTLNDLNETL	0.92	13
ORF1ab1387-2800aa-82	2464	2477	nsp3	FISDEVARDLSLQF	0.92	/
S-61	221	234	S	SALEPLVDLPIGIN	0.92	/
ORF1ab1-1400aa-82	1117	1130	nsp3	VVGPNVNKGEDIQL	0.93	1
ORF1ab1-1400aa-83	478	491	nsp2	ASFSASTSAFVETV	0.93	2
ORF1ab1-1400aa-84	1045	1058	nsp3	IVEEAKKVKPTVVV	0.93	1
ORF1ab2787-4200aa-120	3912	3925	nsp7	VSLLSVLLSMQGAV	0.93	6
ORF1ab2787-4200aa-121	3034	3047	nsp4	LIQPIGALDISASI	0.93	5
ORF1ab5783-7096aa-77	7059	7072	nsp16	SLKEGQINDMILSL	0.93	15
ORF1ab1-1400aa-85	462	475	nsp2	NIVGDFKLNEEIAI	0.94	2
ORF1ab1387-2800aa-83	2387	2400	nsp3	FASFYYVWKSYVHV	0.94	14
ORF1ab1387-2800aa-84	2366	2379	nsp3	WLIINLVQMAPISA	0.94	14
ORF1ab4397-5796aa-70	5445	5458	nsp13	ILANTCTERLKLFA	0.94	16
ORF1ab4397-5796aa-71	5216	5229	nsp12	DDYVYLPYPDPSRI	0.94	18
ORF1ab5783-7096aa-143	6206	6219	nsp14	AVHECFVKRVDWTI	0.94	/
ORF7a-9	103	116	ORF7a	IVAAIVFITLCFTL	0.94	25
ORF1ab1-1400aa-86	1039	1052	nsp3	YIKNADIVEEAKKV	0.95	4
ORF1ab1387-2800aa-85	2346	2359	nsp3	IMQLFFSYFAVHFI	0.95	14
ORF1ab2787-4200aa-122	3943	3956	nsp8	AIASEFSSLPSYAA	0.95	19
ORF1ab2787-4200aa-123	3914	3927	nsp8	LLSVLLSMQGAVDI	0.95	19
ORF1ab4187-4405aa-6	4224	4237	nsp9	KVKYLYFIKGLNNL	0.95	9
ORF1ab4397-5796aa-72	5415	5428	nsp13	GLYKNTCVGSDNVT	0.95	20

Epitope	Start in ORF	End in ORF	Protein	Sequence	Rank	Pool
ORF1ab4397-5796aa-73	4855	4868	nsp12	MCDIRQLLFVVEVV	0.95	17
S-62	609	622	S	AVLYQDVNCTEVPV	0.95	/
S-63	857	870	S	GLTVLPPLLTDEMI	0.95	/
ORF1ab1-1400aa-87	989	1002	nsp3	GQQDGSEDNQTTTI	0.96	/
ORF1ab1-1400aa-88	336	349	nsp2	FVKATCEFCGTENL	0.96	/
ORF1ab1387-2800aa-86	2741	2754	nsp3	KLTCATTRQVVNVV	0.96	14
ORF1ab4187-4405aa-7	4215	4228	nsp9	FVTDTPKGPKVKYL	0.96	/
ORF1ab5783-7096aa-144	6806	6819	nsp16	GVAMPNLYKMQRML	0.96	/
ORF7a-10	75	88	ORF7a	YQLRARSVSPKLFI	0.96	/
S-64	210	223	S	INLVRDLPQGFSAL	0.96	20
M-15	158	171	M	RCDIKDLPKEITVA	0.97	26
ORF1ab2787-4200aa-124	3081	3094	nsp4	NTLLFLMSFTVLCL	0.97	6
ORF1ab2787-4200aa-125	3268	3281	nsp5	KMAFPSGKVEGCMV	0.97	9
ORF1ab2787-4200aa-126	3325	3338	nsp5	SNHNFLVQAGNVQL	0.97	8
ORF1ab2787-4200aa-127	4055	4068	nsp8	GCVPLNIIPLTTAA	0.97	21
ORF1ab2787-4200aa-37	3085	3098	nsp4	FLMSFTVLCLTPVY	0.97	/
ORF1ab4397-5796aa-3	4510	4523	nsp12	RLTKYTMADLVYAL	0.97	21
ORF1ab5783-7096aa-145	6470	6483	nsp15	QQGEVPVSIINNTV	0.97	20
ORF6-4	2	15	ORF6	FHLVDFQVTIAEIL	0.97	25
ORF7a-11	80	93	ORF7a	RSVSPKLFIRQEEV	0.97	/
ORF8-3	108	121	ORF8	FLEYHDVRVVLDFI	0.97	/
S-65	6	19	S	VLLPLVSSQCVNLT	0.97	/
ORF1ab2787-4200aa-128	3452	3465	nsp5	QTAQAAGTDTTITV	0.98	21
ORF1ab5783-7096aa-146	6412	6425	nsp14	HANEYRLYLDAYNM	0.98	16
ORF7a-13	16	29	ORF7a	ELYHYQECVRGTTV	0.98	/
ORF1ab1-1400aa-89	778	791	nsp2	LVGTPVCINGLMLL	0.99	/
ORF1ab2787-4200aa-129	2852	2865	nsp4	PLIAAVITREVGFV	0.99	8
ORF1ab2787-4200aa-130	3462	3475	nsp5	TITVNVLAWLYAAV	0.99	9
ORF1ab4397-5796aa-74	5618	5631	nsp13	GLALYYPSARIVYT	0.99	15
S-66	655	668	S	HVNNSYECDIPIGA	0.99	22
S-67	925	938	S	NQFNSAIGKIQDSL	0.99	/
ORF1ab1-1400aa-90	136	149	nsp2	YGADLKSFDLGDEL	1	/
ORF1ab1387-2800aa-87	2205	2218	nsp3	SVGKFCLEASFNYL	1	14
ORF1ab1387-2800aa-88	1654	1667	nsp3	KKWKYPQVNGLTSI	1	13
ORF1ab2787-4200aa-131	3809	3822	nsp6	GVYDYLVSTQEFRY	1	5
ORF1ab2787-4200aa-132	3842	3855	nsp6	IKLLGVGGKPCIKV	1	8
ORF1ab4397-5796aa-75	5702	5715	nsp13	MATNYDLSVVNARL	1	17
ORF1ab4397-5796aa-76	5118	5131	nsp12	RLYECLYRNRDVDT	1	18
ORF1ab5783-7096aa-147	6979	6992	nsp16	YKLMGHFAWWTAFV	1	/
S-68	946	959	S	GKLQDVVNQNAQAL	1	22
ORF1ab2787-4200aa-5	2901	2914	nsp4	KLIEYTDFATSACV	1.1	6
S-2	222	235	S	ALEPLVDLPIGINI	1.1	21
S-3	976	989	S	VLNDILSRLDKVEA	1.1	12

Epitope	Start in ORF	End in ORF	Protein	Sequence	Rank	Pool
S-4	584	597	S	ILDITPCSFGGVSV	1.1	3
ORF1ab1387-2800aa-5	1675	1688	nsp3	YLATALLTLQQIEL	1.2	/
ORF1ab2787-4200aa-6	4089	4102	nsp8	FTYASALWEIQQVV	1.2	6
ORF1ab2787-4200aa-7	3248	3261	nsp4	VLYQPPQTSITSAV	1.2	6
ORF1ab4397-5796aa-4	4918	4931	nsp12	ALFAYTKRNVIPTI	1.2	21
ORF1ab5783-7096aa-78	6165	6178	nsp14	FMIDVQQWGFTGNL	1.2	23
S-5	995	1008	S	RLITGRLQSLQTYV	1.2	22
ORF1ab1-1400aa-3	440	453	nsp2	GEGSEGLNDNLLEI	1.3	1
N28274-29533-2	217	230	N	AALALLLLDRLNQL	1.4	26
ORF1ab1387-2800aa-6	2294	2307	nsp3	GLDSLDTYPSLETI	1.4	/
ORF1ab2787-4200aa-8	3111	3124	nsp4	YLTFYLTNDVSFLA	1.4	14
ORF1ab5783-7096aa-79	6073	6086	nsp14	HLIPLMYKGLPWNV	1.4	15
ORF1ab1-1400aa-4	1285	1298	nsp3	YIVGDVVQEGVLTA	1.5	2
ORF1ab2787-4200aa-10	3705	3718	nsp6	ARRVWTLMNVLTLV	1.5	9
ORF1ab2787-4200aa-11	2956	2969	nsp4	LMDGSIIQFPNTYL	1.5	6
ORF1ab2787-4200aa-9	2962	2975	nsp4	IQFPNTYLEGSVRV	1.5	6
S-6	971	984	S	GAISSVLNDILSRL	1.5	20
ORF1ab4397-5796aa-5	5229	5242	nsp12	ILGAGCFVDDIVKT	1.6	15
ORF1ab1-1400aa-5	1354	1367	nsp3	YILPSIISNEKQEI	1.7	/
ORF1ab1387-2800aa-7	1437	1450	nsp3	TLNDLNETLVTMPL	1.7	13
ORF1ab1387-2800aa-8	1433	1446	nsp3	SLINTLNDLNETLV	1.7	/
ORF1ab1387-2800aa-9	1734	1747	nsp3	YLFQHANLDSCKRV	1.7	/
ORF1ab2787-4200aa-12	4044	4057	nsp8	ALNNIINNARDGCV	1.7	6
ORF1ab4397-5796aa-6	4451	4464	nsp12	KDEDDNLIDSYFVV	1.7	20
ORF1ab5783-7096aa-80	6903	6916	nsp16	SDADSTLIGDCATV	1.7	23
ORF3a-3	184	197	ORF3a	YQIGGYTEKWESGV	1.7	24
N28274-29533-3	112	125	N	YLGTGPEAGLPYGA	1.8	26
ORF1ab1-1400aa-6	1333	1346	nsp3	GLNGYTVEEAKTVL	1.8	/
ORF1ab2787-4200aa-13	3639	3652	nsp6	FLLPSLATVAYFNM	1.8	/
ORF1ab2787-4200aa-14	3917	3930	nsp7	VLLSMQGAVDINKL	1.8	14
ORF1ab4397-5796aa-7	5235	5248	nsp12	FVDDIVKTDGTLMI	1.8	15
ORF1ab5783-7096aa-81	6523	6536	nsp15	ILNNLGVDIAANTV	1.8	17
ORF1ab5783-7096aa-82	6096	6109	nsp14	SDTLKNLSDRVVFV	1.8	17
S-7	1048	1061	S	HLMSFPQSAPHGVV	1.8	3
ORF1ab1387-2800aa-10	1663	1676	nsp3	GLTSIKWADNNCYL	1.9	/
ORF1ab2787-4200aa-15	3822	3835	nsp6	YMNSQGLLPPKNSI	1.9	14
ORF1ab4397-5796aa-8	4632	4645	nsp12	LLMPILTLTRALTA	1.9	15
ORF1ab5783-7096aa-83	6333	6346	nsp14	NLNLPGCDGGSLYV	1.9	23
ORF3a-4	114	127	ORF3a	FLQSINFVRIIMRL	1.9	/
E-1	4	17	E	FVSEETGTLIVNSV	2	25
ORF1ab1387-2800aa-11	1957	1970	nsp3	VTFFPDLNGDVVAI	2	/
ORF1ab2787-4200aa-16	3512	3525	nsp5	ILGPLSAQTGIAVL	2	/
ORF1ab2787-4200aa-17	3064	3077	nsp4	FMRFRRAFGEYSHV	2	5

Epitope	Start in ORF	End in ORF	Protein	Sequence	Rank	Pool
ORF1ab2787-4200aa-18	3178	3191	nsp4	AALCTFLLNKEMYL	2	9
ORF1ab5783-7096aa-84	6796	6809	nsp15	KLQSSQAWQPGVAM	2	23
S-8	980	993	S	ILSRLDKVEAEVQI	2	22

Assigned epitope name, start and end positions within the corresponding ORF (denoted at the start of epitope name), protein end product it is located in, epitope amino acid sequences, percentile ranks calculated by IEDB prediction algorithm, and the peptide pool it was included in for initial PBMC stimulation (/, epitope peptide synthesis failure) are listed.

Supplementary Table S2. Distribution of predicted HLA-A*02:01-restricted CD8^{+} epitopes in SARS-CoV-2-encoded proteins

Protein (length, location within ORF)	Epitopes predicted	Protein length encompassed	Epitopes synthesized	Epitopes recognized by healthy donor PBMC	Epitopes recognized by convalescent patient PBMC
nsp1 (180 aa, ORF1ab 1-180)	15	60%	15	0	1
nsp2 (638 aa, ORF1ab 181-818)	36	53%	32	0	3
nsp3 (1945 aa, ORF1ab 819-2763)	121	53%	99	1	5
nsp4 (500 aa, ORF1ab 2764-3263)	49	65%	46	0	5
nsp5 (306 aa, ORF1ab 3264-3569)	18	56%	17	0	6
nsp6 (290 aa, ORF1ab 3570-3859)	35	81%	34	0	4
nsp7 (83 aa, ORF1ab 3860-3942)	12	96%	12	0	3
nsp8 (198 aa, ORF1ab 3943-4140)	17	66%	17	0	1
nsp9 (113 aa, ORF1ab 4141-4253)	6	54%	5	0	2
nsp10 (139 aa, ORF1ab 4254-4392)	4	33%	3	0	0
nsp12 (932 aa, ORF1ab 4393-5324)	49	49%	49	0	0
nsp13 (601 aa, ORF1ab 5325-5925)	32	55%	31	0	1
nsp14 (527 aa, ORF1ab 5926-6452)	23	39%	19	0	0
nsp15 (346 aa, ORF1ab 6453-6798)	22	63%	16	0	0
nsp16 (298 aa, ORF1ab 6799-7096)	16	63%	8	0	0
E (75 aa)	9	89%	9	0	0
M (222 aa)	15	55%	13	0	1
N (419 aa)	12	34%	11	0	1
S (1273 aa)	63	49%	57	0	0
ORF3a (275 aa)	24	66%	19	0	0
ORF6 (61 aa)	4	61%	4	0	0
ORF7a (121 aa)	12	79%	4	0	0
ORF7b (43 aa)	5	88%	2	0	0
ORF8 (121 aa)	3	27%	1	0	0
ORF10 (38 aa)	2	74%	1	0	0

Supplementary Table S3. Clinical profile of convalescent patients

Patient	Age	Gender	Date of admission	Date of discharge	Symptoms
**A**	42	male	2020.01.28	2020.02.08	mild
**B**	30	male	2020.02.07	2020.02.14	severe
**C**	28	female	2020.02.08	2020.02.18	severe
**D**	39	male	2020.02.07	2020.02.14	mild
**E**	51	female	2020.02.13	2020.02.19	mild
**F**	30	male	2020.02.08	2020.02.16	mild
**G**	56	male	2020.01.20	2020.01.27	severe
**H**	53	female	2020.01.20	2020.01.27	severe
**I**	59	male	2020.02.08	2020.02.18	severe
**J**	66	male	2020.01.26	2020.02.20	severe
**K**	32	male	2020.01.21	2020.01.29	mild
**L**	59	female	2020.02.06	2020.02.15	severe
**M**	41	male	2020.02.06	2020.02.20	mild
**N**	43	male	2020.01.20	2020.02.24	mild
**O**	46	female	2020.01.27	2020.02.18	mild
**P**	29	male	2020.01.27	2020.02.08	severe
**Q**	41	female	2020.01.28	2020.02.16	severe
**R**	56	male	2020.01.26	2020.02.21	severe
**S**	77	female	2020.01.26	2020.02.01	severe
**T**	49	male	2020.01.30	2020.02.18	severe

Supplementary Table S4. Epitope recognized by PBMC from healthy donors

Epitope	Start in ORF	End in ORF	Protein	Sequence	Rank	Sample that recognized the epitope
ORF1ab2787-4200aa-73	3843	3856	nsp6	KLLGVGGKPCIKVA	0.59	0910B
